# Systems analysis shows that thermodynamic physiological and pharmacological fundamentals drive COVID‐19 and response to treatment

**DOI:** 10.1002/prp2.922

**Published:** 2022-02-01

**Authors:** Richard J. Head, Eugenie R. Lumbers, Bevyn Jarrott, Felix Tretter, Gary Smith, Kirsty G. Pringle, Saiful Islam, Jennifer H. Martin

**Affiliations:** ^1^ Drug Discovery and Development, Clinical and Health Sciences University of South Australia Adelaide South Australia Australia; ^2^ School of Biomedical Sciences & Pharmacy University of Newcastle Newcastle New South Wales Australia; ^3^ Hunter Medical Research Institute New Lambton Heights New South Wales Australia; ^4^ Florey Institute of Neuroscience & Mental Health University of Melbourne Parkville Victoria Australia; ^5^ Bertalanffy Center for the Study of Systems Science Vienna Austria; ^6^ VP System Practice ‐ International Society for System Sciences Pontypool UK; ^7^ Centre for Drug Repurposing and Medicines Research Clinical Pharmacology University of Newcastle Newcastle New South Wales Australia

**Keywords:** ACE2, affinity, complex systems, COVID‐19, dissociation constant, Gibbs free energy, innate immunity, kinetics, law of mass action, MERS, pandemic, pharmacology, pharmacology, physiology, physiology, renin angiotensin system, SARS‐CoV‐1, SARS‐CoV‐2, thermodynamics, tropism, variants, virus

## Abstract

***Why a systems analysis view of this pandemic?*** The current pandemic has inflicted almost unimaginable grief, sorrow, loss, and terror at a global scale. One of the great ironies with the COVID‐19 pandemic, particularly early on, is counter intuitive. The speed at which specialized basic and clinical sciences described the details of the damage to humans in COVID‐19 disease has been impressive. Equally, the development of vaccines in an amazingly short time interval has been extraordinary. However, what has been less well understood has been the fundamental elements that underpin the progression of COVID‐19 in an individual and in populations. We have used systems analysis approaches with human physiology and pharmacology to explore the fundamental underpinnings of COVID‐19 disease. Pharmacology powerfully captures the thermodynamic characteristics of molecular binding with an exogenous entity such as a virus and its consequences on the living processes well described by human physiology. Thus, we have documented the passage of SARS‐CoV‐2 from infection of a single cell to species jump, to tropism, variant emergence and widespread population infection. During the course of this review, the recurrent observation was the efficiency and simplicity of one critical function of this virus. The lethality of SARS‐CoV‐2 is due primarily to its ability to possess and use a variable surface for binding to a specific human target with high affinity. This binding liberates Gibbs free energy (GFE) such that it satisfies the criteria for thermodynamic spontaneity. Its binding is the prelude to human host cellular entry and replication by the appropriation of host cell constituent molecules that have been produced with a prior energy investment by the host cell. It is also a binding that permits viral tropism to lead to high levels of distribution across populations with newly formed virions. This thermodynamic spontaneity is repeated endlessly as infection of a single host cell spreads to bystander cells, to tissues, to humans in close proximity and then to global populations. The principal antagonism of this process comes from SARS‐CoV‐2 itself, with its relentless changing of its viral surface configuration, associated with the inevitable emergence of variants better configured to resist immune sequestration and importantly with a greater affinity for the host target and higher infectivity. The great value of this physiological and pharmacological perspective is that it reveals the fundamental thermodynamic underpinnings of SARS‐CoV‐2 infection.

## Graphical abstract

The lethality of SARS‐CoV‐2 is due primarily to its ability to possess and utilize a variable surface for binding to a specific human target with high affinity. This binding liberates Gibbs free energy such that it satisfies the criteria for thermodynamic spontaneity. A unique synchrony of viral surface change with Gibbs free energy liberation.

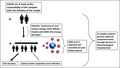

**Abbreviations**
ACE2: angiotensin‐converting enzyme 2
GFE: Gibbs free energy
HA: haemagglutinin
IFN‐α: interferon‐alpha
ISG: interferon‐stimulated gene
MERS: Middle East Respiratory Syndrome
NALT: nasopharyngeal‐associated lymphoid tissue
PCL: periciliary layer
PRR: pattern recognition receptor
RBD: receptor‐binding domain
SARS: severe acute respiratory syndrome

## 1 BACKGROUND

This pandemic is an example of a complex systems analysis of the devasting intersection of human biological complexity with severe acute respiratory syndrome (SARS)‐CoV‐2 lethal simplicity. To better understand this intersection, there is a need to view this viral infection not from the perspective of the diverse disciplines underpinning medicine in isolation^1^ but rather from the standpoint of an evolving series of physical and biological transformations in a fashion similar to that described by Trancossi et al.^2^ Experimentally these transformations will depend on an understanding of the contribution of not from single cells but from appropriate multiple cell types and tissues in an integrated fashion. The importance of the underlying developmental biology has been highlighted recently by Chen et al.^3^

### 1.1 The vulnerability of the complex

*Homo sapiens* is a successful multicellular natural complex of systems. The success in complex systems is the essential design patterns that function to compete, survive, reproduce, and evolve over multiple generations toward fitness and growth.^4^ A major design pattern in human development has been the devolution and automation of thousands of systemic processes to leave the brain free from overburdening decision‐making, to leave it uncluttered. For this purpose, the common design pattern uses mechanisms in automation based on a binary balance/counter‐balance process. The cornerstone of human physiology is this design that regulates and moderates minute to minute changes in an autonomous manner for complex systems that interact with their environments through sensors and actuators.^4^

Although this essential design pattern for autonomous function in the human is efficient, it is vulnerable to dysregulation by pathogens that convert sophisticated binary regulation into a destructive nonbinary state. This is the essence of the pathophysiology of COVID‐19. Dysregulation of balance/counterbalance states is not unique to COVID‐19 disease. For example, the life‐threatening disruption of colonic balance of chloride and sodium ions in cholera is driven by the continuous stimulation of adenylate cyclase and G protein interaction mediated by a toxin from the bacterium *Vibrio cholerae*. A microbial toxin induced shift from a binary regulated state. This principle of dysregulation underpinning serious disease states occurs in noninfectious settings, e.g. cancer. The integrity of the DNA structure is vital for genomic stability and achieved with a balance between DNA repair, checkpoint arrest, apoptosis, cell cycle arrest, and replication. In cancer cells, either downregulation or mutations in the repair genes compromise the genomic stability by by‐passing the binary regulatory processes.

**FIGURE 1:**
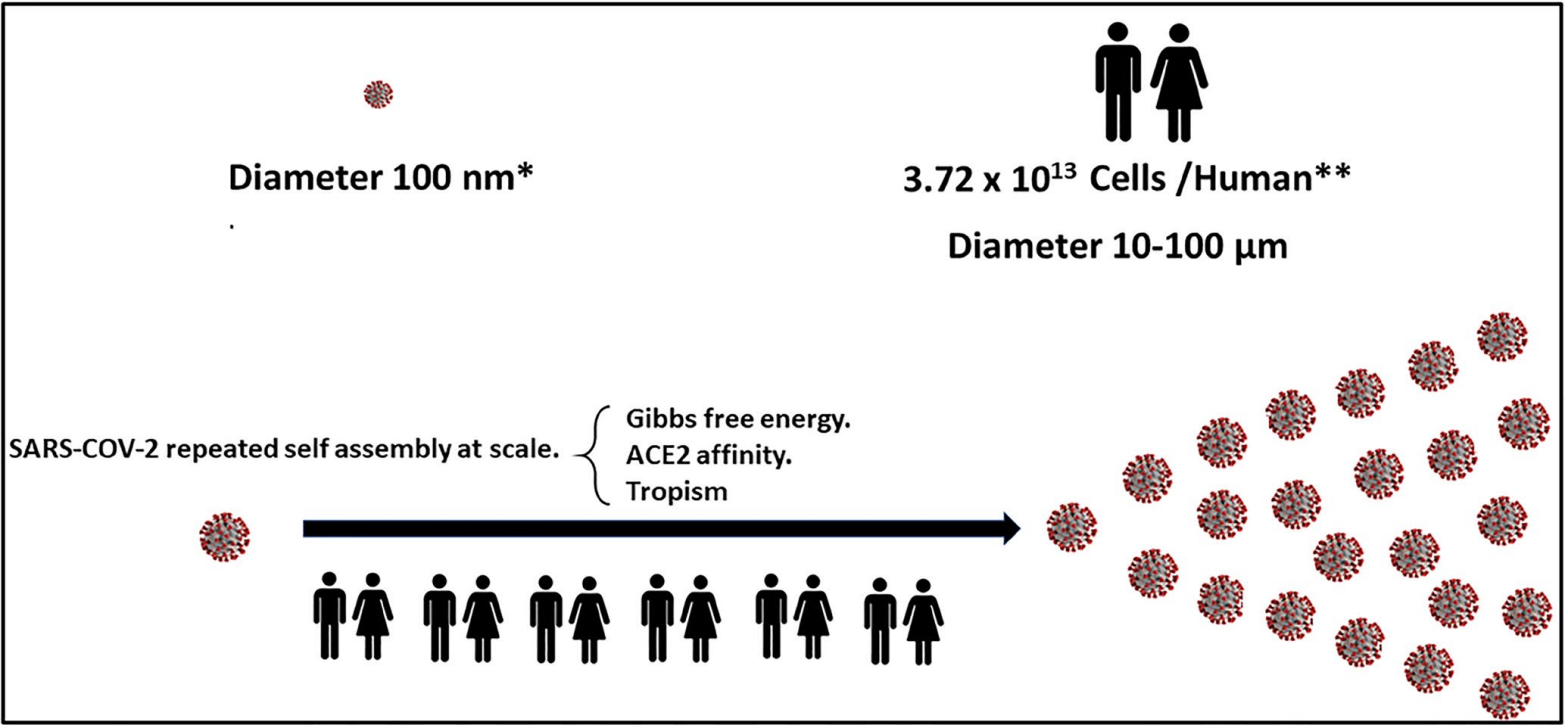
Repeated SARS‐CoV‐2 self‐assembly at scale drives the vulnerability of the complex and lethality of the simple. Illustration of the difference in scale and hence complexity between a human cell and a single SARS‐CoV‐2 virion. It shows that populations provide the platform to facilitate the repeated self‐assembly at scale of SARS‐CoV‐2. The number of cells estimated to be present in a human is of the order of 37 trillion.^8^ The diameter of a single SARS‐CoV‐2 is approximately 100 nm^9^ or about a 1000 times smaller than the diameter of an average human cell (100 µm)

Prior to the current pandemic, complex systems approaches had been described for infectious disease surveillance and response in which the importance of systems modelling was stressed in predicting temporal spatial patterns as well as identifying underlying interactions as a key to decision‐making.^5^ It is these underlying interactions involving physicochemical fundamental principles that underpin the pathophysiology of infection with SARS‐CoV‐2. Events that provide the platform for SARS‐CoV‐2 to drive COVID‐19 are in a physical/chemical interplay between the complex and the simple (Figure 1).

### 1.2 The lethality of the simple

In viewing the current pandemic, one is drawn to the prevailing lethality of the simple. SARS‐CoV‐2 is constrained with few fundamental processes to drive its success in this pandemic. Systems thinking seeks to define what it is that is commonly used and repeated as the virus successfully progresses from a vector to a human, from cell to cell, organ to organ, and human to human. The lethality of SARS‐CoV‐2 relates, with simplicity, to a small but changeable viral surface architecture that triggers entry into the host cell, evasion of host defenses and subsequent replication. Specifically, it is the high‐affinity interaction between the receptor‐binding domain (RBD) of the SARS‐CoV‐2 spike protein and the host cell angiotensin‐converting enzyme 2 (ACE2) protein. It is the repeated SARS‐CoV‐2 occupancy of human ACE2 (hACE2) molecular sites together with the power of tropism, a biological effect causing a directed response, in this case the laws of thermodynamics and receptor affinity, that drives COVID‐19 disease (Figure 1). A frequent process in biology involving molecular recognition in which biological macro molecules form specific ligand interactions, comprehensively described as by Du et al.^6^ and detailed in a similar fashion by Sepahvandi et al.^7^ for SARS‐CoV‐ 2 in COVID‐19 disease.

This manuscript will focus on both the vulnerability of the complex and lethality of the simple to outline the key intersections of basic ways of energy, chemistry and pharmacology that enable successful viral entry and replication.

## 2 WHAT DRIVES VIRAL ENTRY?

Contrary to common thinking, ACE2 is not only the entry point for SARS‐CoV‐2 into host cells but the key to understanding SARS‐CoV‐2 pathogenesis.^5^ It is an entry event facilitated by the fundamentals of thermodynamics and its attending laws of mass action; an entry facilitated by the architectural change in the surface of SARS‐CoV‐2 that evades sequestration from the host's immune armamentarium, and which, on binding to the host, unleashes a shift in entropy and enthalpy to initiate a thermodynamically spontaneous event.

### 2.1 The thermodynamics, kinetics, and SARS‐CoV‐2

SARS‐CoV‐2 is an obligate and, as such, is incapable of reproduction on its own. It is devoid of metabolic activity and depends on appropriating host cellular processes for self‐assembly and release into the extracellular medium. It has been established for decades that ligand–receptor interaction and stabilization by enthalpy and/or entropy can be analyzed by thermodynamics.^10^ It is, therefore, not unreasonable to view the entire process of replication and self‐assembly of SARS‐CoV‐2 from the standpoint of the fundamental laws of physics and chemistry. This is consistent with the view that thermodynamic studies are increasingly important in understanding the biological environment.^7^ In a comprehensive analysis, Du et al. have drawn attention to Gibbs free energy (GFE) being the thermodynamic potential for characterizing driving force and the degree of protein–ligand association, which is determined by the extent of the negative GFE.^6^

**FIGURE 2:**
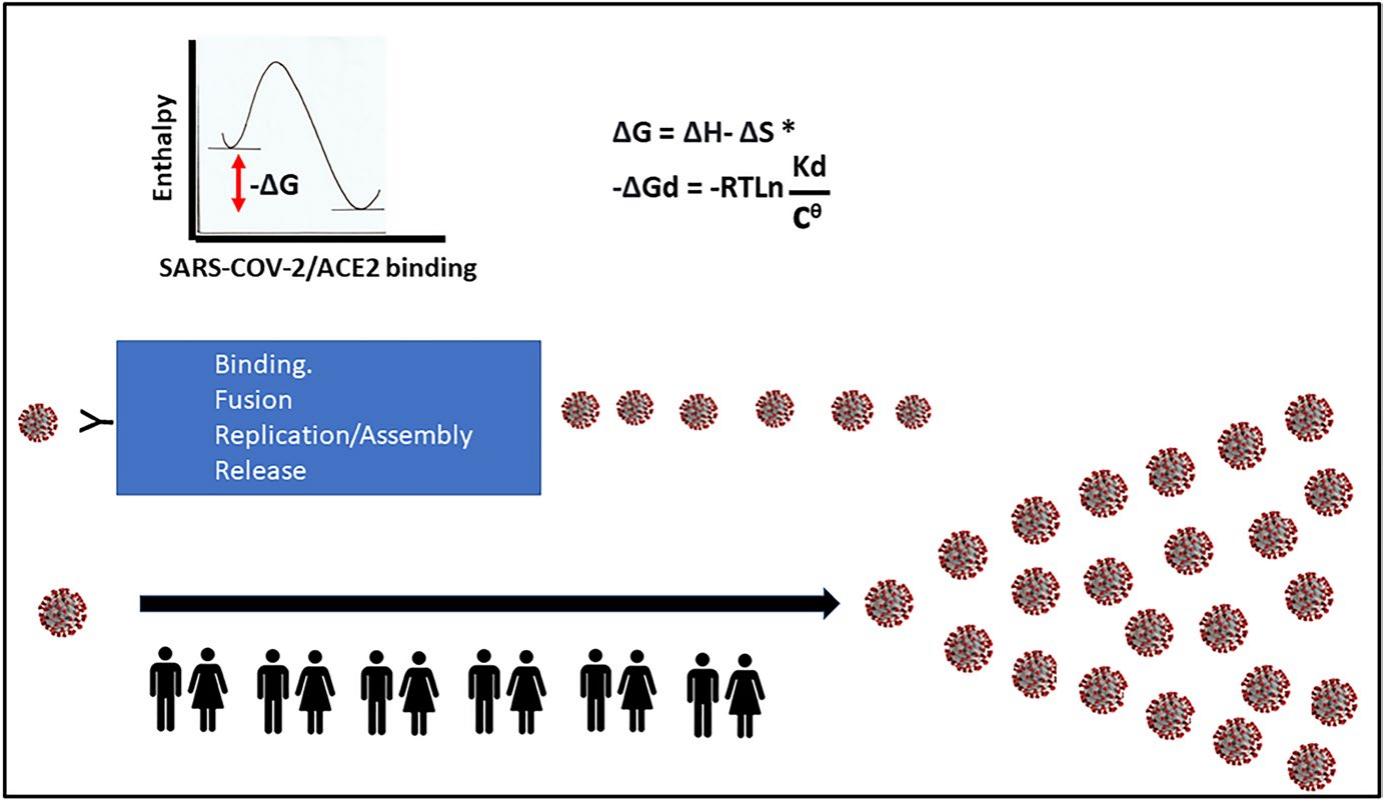
SARS‐CoV‐2 repeated self‐assembly using thermodynamic spontaneity. The repeated self‐assembly of SARS‐CoV‐2 is driven by Gibbs free energy (GFE), and it is this useable energy that predicts spontaneous change. Shown is the relationship between the dissociation constant (K_d_) and the change in GFE of dissociation.^7^ The negative GFE for binding of SARS‐CoV‐2 to ACE2 is illustrated. The GFE of the growth of the nucleocapsids for SARS‐CoV‐2 is more negative than that of the host tissue.^12^ Illustrated is the view that populations provide the platform to facilitate the repeated self‐assembly at scale of SARS‐CoV‐2. (ΔGd—GFE of dissociation, ΔG—Change in GFE, ΔH—change in enthalpy, ΔS—change in entropy, Kd—dissociation constant, R—ideal gas constant, T—absolute temperature, cθ = 1 mol/L)

Bruinsma et al. have drawn attention to the fact that viral capsid assembly adheres to the law of mass action in chemical thermodynamics.^11^ For example, it is the thermodynamic potentials that describe how a virus adsorbs to its host receptor.^7^ Of importance in biological systems is the estimate of GFE—usable energy that predicts the spontaneous change. The change in GFE depends on the prevailing energy of the products and reactants and when negative, indicates a spontaneous reaction that is thermodynamically possible (Figure 2).

As highlighted recently by Popovic and Minceva, viral multiplication is a chemical process and through nonequilibrium thermodynamics permits GFE of growth comparative analysis.^12^ In view of the effectiveness of SARS‐CoV‐2 in binding to hACE2 and subsequent entry into cells, viral replication and release, the entire process can be viewed in thermodynamic terms as an exergonic reaction. This follows from the view that thermodynamic potentials describe how a virus adsorbs to a host receptor^7^ and that in coronaviruses it is the free energy released upon subsequent refolding of the fusion protein to its most stable conformation that facilitates the close apposition of viral and cellular membranes and the actual membrane merger.^13^ More recently, Popovic and Minceva in describing the three corona viruses SARS‐CoV‐1, Middle East Respiratory Syndrome (MERS), and SARS‐CoV‐2, highlighted that empirical formulas permitted calculation of the thermodynamic properties of the viruses for formation and growth and that GFE permits an estimate of the spontaneity of new virion formation.^12^ In other words, infection by SARS‐CoV‐2 may be able to be explained as a nonequilibrium event characterized by an overall negative change in GFE and is as such spontaneous (Figure 2).

## 3 COVID‐19—A DISEASE DRIVEN BY THERMODYNAMIC SPONTANEITY AND MASS ACTION

As highlighted by Trancossi et al., a virus has more negative GFE than its host and this is a critical element in infection.^2^ The discussion below explores the possibility that the SARS‐CoV‐2 surface architecture, its RBD together with its interaction with the host target protein (ACE2) drives the spontaneity for adhesion, entry, replication, and efflux in a thermodynamically acceptable path that powers COVID‐19 disease (Figure 2).

### 3.1 Gibbs free energy of growth and intracellular SARS‐CoV‐2 replication

It is convenient from a thermodynamic perspective to view the processes of SARS‐CoV‐2 binding to its receptor and the process of subsequent replication as linked discrete processes. In that regard, Popovic and Minceva demonstrated that the GFE of growth of the nucleocapsids of SARS, MERS, and SARS‐CoV‐2 was more negative than that of the host tissue.^12^ The greater the negativity of the GFE of growth, the greater the spontaneous virus multiplication using host cell synthetic processes is. The new viral replication, assembly and release is also, in thermodynamic terms, biased in favor of the virus as an obligate parasite. It is noteworthy that a virus has no metabolic systems to generate energy and, in the absence of a host exhibits no energy flux and therefore there is no GFE change. However, when a virus binds to a host's surface proteins then the attending shift in enthalpy and entropy is associated with a negative GFE response. Popovic and Minceva hypothesized that the GFE for the virus is always more negative than the GFE of the host suggesting that the synthesis of viral components is favored thermodynamically^12^ (Figure 2).

The viral appropriation of the host cellular machinery is also made possible because of the prior energy costs borne by the infected cell in the provision of the cellular accoutrements for viral assembly and release. It can be concluded that this aspect of the cellular infection process obeys nonequilibrium dynamics. It is nonreversible and importantly, based on GFE considerations, is spontaneous in nature. What is fundamental is that this thermodynamically spontaneous multiplication of SARS‐CoV‐2 is only initiated after the virus binds to the host ACE2 protein, which facilitates cellular entry.

### 3.2 Gibbs free energy of growth and SARS‐CoV‐2 receptor binding

An enormous amount of information regarding the function and structure of the spike protein has been accumulated and published as this pandemic has progressed, and there are excellent descriptive summary texts accompanied with informative illustrations. By way of summary, the key to infection of humans by SARS‐CoV‐2 is the role of the spike glycoprotein that binds to the ACE2 on the surface of targeted cells. The spike protein is comprised of two key subunits–one (S1) that houses the RBD and the other (S2) that orchestrates the binding of the virus to the host lipid membrane. Of fundamental importance is the binding affinity of the RBD for ACE2, which is related to Van der Waals interactions the electrostatic properties and the polar and nonpolar amino acid interactions. These are well described in Sepahvandi et al.^7^

As highlighted by Sepahvandi et al., it is commonly thought that kinetics and thermodynamics influence the virus interaction with host receptors in as much as the thermodynamic potentials describe the adsorption of the virus onto the host receptor.^7^ The forces that drive these molecular associations require measurement of changes of key thermodynamic parameters, including free energy of binding (Δ*G*), enthalpy (Δ*H*), and entropy (Δ*S*) of binding.^14^ Of critical importance is the virus–host receptor interaction viewed initially as a reversible process the strength of which is defined as the binding affinity. As illustrated by Sepahvandi et al. the physicochemical thermodynamic description of binding affinity is translated as the dissociation constant (Kd), whereas the physical thermodynamic description is translated into the GFE of dissociation (ΔGd).^7^

Details on the binding of SARS‐CoV‐2 to ACE2 are well documented and importantly can be interpreted in thermodynamic terms. As mentioned earlier, the thermodynamic interactions commence with the levels of energy of the reactants. During the binding of the S protein of SARS‐CoV‐2 to ACE2, the change in adsorption enthalpy reflects the sum of bond energy changes which determines the change in GFE.^7^ When the GFE is negative, the thermodynamic considerations indicate this will be a spontaneous reaction. Of significance is the observation that the estimated binding energies for the spike protein structures of SARS‐CoV‐2 and SARS‐CoV are both negative and the lowest binding energy value is associated with SARS‐CoV‐2.^15^ Additionally, several mutations in the receptor‐binding motif for SARS‐CoV‐2 lead to this change in binding energy when SARS‐CoV‐2 is compared with SARS‐CoV.^15^ In a detailed study, Koley et al. conducted sequence and structure‐based analysis with SARS‐CoV‐2 spike RBD complex and hACE2 and reported a negative binding energy value.^16^ Moreover, they compared the GFE of binding across a range of mammalian complexes using the human as a reference standard, demonstrating lower affinity between SARS‐CoV‐2 spike protein affinity and ACE2 from other mammalian species.^16^ In a similar fashion, Piplani et al. also examined the binding free energies of SARS‐CoV‐2 spike protein across a range of species and demonstrated they were negative, supporting the view that the binding of SARS‐CoV‐2 spike protein to hACE2 was higher than any other species examined.^17^ It also suggested that for ACE2 species within an upper affinity range, a correlation exists between binding free energies and infectivity, a fact critical in understanding the effect of mutations and contemplating pharmacological strategies to halt the infection.

### 3.3 Viral binding—The trigger for host cell infection facilitated by Gibbs free energy of growth

The interaction between a virus and host cell involves an interaction of thermodynamic evolution with time.^2^ It follows that for SARS‐CoV‐2 infection, the adsorption of the spike protein to the ACE2 receptor is the initiating event necessary for subsequent cellular replication of the virus. The synchrony between binding and subsequent replication drives the host cell infection. The two critical elements in that process are both characterized by negative GFE and as such are thermodynamically spontaneous. It has been proposed that there is a greater spontaneous virus multiplication rate which produces an enhanced reservoir of virus and permits greater population transmission.^12^ It is the thermodynamics that is likely to determine much of the binding of the SARS‐CoV‐2 to the host. However, it is the negative GFE that determines the stability of the protein‐ligand complex and, as such, the binding affinity of a ligand to a receptor.^6^ It is this binding energy that is converted to the dissociation constant, measured by the binding of the spike protein for ACE2.^7^ As highlighted by Du et al., the ratio of the kinetic parameters (k on and k off) determines the thermodynamic properties including the stability of the complex and the binding affinity between the protein and ligand.^6^ In this way, the thermodynamic nature (GFE) of the adsorption of SARS‐CoV‐2 to the receptor domain of ACE2 is integrated with the rate kinetics embodied in the law of mass action.

### 3.4 From Gibbs energy of activation to dissociation constant for SARS‐CoV‐2—A fundamental measure of infectivity and progression of COVID‐19 disease

Thermodynamics describes how SARS‐CoV‐2 binds to hACE2 and kinetics describes the rate at which SARS‐CoV‐2 comes into contact with the binding sites on hACE2. The binding affinity (dissociation constant) is translated into the GFE of dissociation. Shang et al. have summarized many of the reported K_D_ values for the binding of the SARS‐CoV‐2 spike protein and the binding of the SARS‐CoV‐2 RBD.^5^ In doing so, they draw attention to the complexity of the SARS‐CoV‐2 spike protein binding that is related to the size and shape of the protein interaction. Regardless, the binding of the spike protein‐binding domain for ACE2 has been well documented,^18^, ^19^ and the K_D_ values for high affinity binding to ACE2 protein were determined to be in the order of 3.0 nM^20^ to 4.6 nM.^19^

The ability to quantify the binding of the SARS‐CoV‐2 spike protein to ACE2, the triggering event for infectivity, is the most powerful measure in COVID‐19 disease for three reasons:

1. *Outcome prediction*. The extent of the initial binding of SARS‐CoV‐2 spike protein to ACE2 establishes the pattern by which subsequent linked processes quantitatively follow. To that extent, it is not unreasonable to assume that the kinetics of the initial binding of SARS‐CoV‐2 to ACE2 obey the laws of mass action and, as such, the kinetics of that initial binding step will predict the cellular and/or tissue potency of this virus. Support for this view comes from a comparison between the binding of SARS‐CoV‐2 and the less infectious SARS‐CoV as it is commonly seen that the affinity of the SARS‐CoV‐2 RBD is greater than that for the SARS‐CoV RBD. This is precisely the same phenomenon of a predictive role of initial ligand binding already demonstrated in the cancer field, where initial in vitro biochemical binding affinities predict the concentration range in which kinase inhibitors will be active in intact cells.^21^ In addition, as discussed by Walls et al., the rate of viral replication in distinct species, transmissibility, and disease severity correlate with the binding affinity of SARS‐CoV‐2 for hACE2.^22^ Moreover, the binding affinity of the spike protein for ACE2 has been suggested to be a major determinant of SARS‐CoV‐2 replication and disease severity.^23^ It is fortuitous that the measurement of affinity by way of the measurement Kd has underpinned drug development for decades and that experience in predicting potency and comparative potencies is transferrable to characterizing the binding of SARS‐CoV‐2 and its variants to the ACE2 receptor.

2. *Quantifying viral tropism and host interaction*. Quantification of the binding of the SARS‐CoV‐2 spike protein to ACE2 is an invaluable measure in understanding the tropism associated with this binding. Piplani et al. showed that across ACE2 from different species (within an upper affinity range for SARS‐CoV‐2 spike protein binding to ACE2), a correlation exists between binding free energies and infectivity.^17^ The concurrent orientation and interaction of the critical binding sites of the host receptor (ACE2) and the virus (SARS‐CoV‐2) are the combined trophic endpoints that permit quantification by way of the dissociation constants.

3. *Predicting the potency of SARS‐CoV‐2 variants*. Mutations in the SARS‐CoV‐2 spike protein can enhance the binding affinity of SARS‐CoV‐2 to ACE2. This is well illustrated for the D614G mutation of the SARS2‐S protein where, due to a slower dissociation constant of this mutant, there is a 1.5‐ or 2‐fold increase in the binding affinity for ACE2, possibly from mutation induced structural flexibility.^24^ Moreover, Ozono et al. conclude that cell entry is enhanced due to this higher affinity. Further support for a relationship between mutations in SARS‐CoV‐2 RBD and binding affinities for ACE2 comes from the studies of Mejdani et al. drawing attention to high‐frequency mutations (S477N, N439K, V367F, and N501Y) that have improved binding affinity to ACE2.^25^ Of particular importance in this pandemic are the observations suggesting that variant spike proteins enhance the transmissibility of B.1.1.7 (alpha variant) and B.1.351 (beta variant) in part by enhancing the affinity of the spike protein variants to ACE2.^26^ The ability to quantify the binding of the SARS‐CoV‐2 spike protein to ACE2 provides the window through which to view the key determinants of COVID‐19 disease.

## 4 THE ROLE OF VIRAL TROPISM

### 4.1 Broad‐based SARS‐CoV‐2 tropism in the human

It is this combination of cellular and host tropism, together with the thermodynamics and kinetics of the binding of SARS‐CoV‐2 to ACE2 that underpins the development of COVID‐19. Previous experience with the influenza A virus illustrates how viruses must change their tropism to preferentially target a new species.^27^ An appreciation of these determinates requires an understanding of the nature of SARS‐CoV‐2 tropism in the human, the molecular basis that defines that viral tropism and the degree to which these structural features provide the platform for thermodynamic spontaneity.

The efficiency of SARS‐CoV‐2 in recognizing binding to a host receptor determines the preference of this virus for a given species, tissue or cell type.^28^ The lethality and targeting of hosts by SARS‐CoV‐2 is driven by simplicity, namely the ability to manipulate and change a very small portion of its viral surface such that it can infect a susceptible host. Rawat et al. demonstrated, in a comparison between three coronaviruses that bind to ACE2, that the interface surface area of the spike protein for the ACE2 complex was smaller for HCoV‐NL63 than that of SARS‐CoV and SARS‐CoV‐2, concluding that the mild HCoV‐NL63 has less binding affinity than the two other strains.^29^ As indicated by Zhang et al. the binding affinities for ACE2 are clinically relevant, in the nanomolar region from 5 to 95 nM.^30^ Additionally, the strong interaction between ACE2 and the SARS‐CoV‐2 spike protein is a characteristic of this virus that is reflected in its high transmissibility rate, infectivity, and global spread.^30^, ^31^

As accentuated by Zhao et al. viral spike protein recognition is the key determinant of the host range.^32^ In a similar fashion, Liu et al. provided data indicating a broad host tropism for SARS‐CoV‐2.^33^ Within humans a significant cellular tropism with SARS‐CoV‐2 exists. Importantly, the hACE2 protein is expressed on many different cell types and thus, ACE2 is present in many organs including blood vessels, the lung, heart, kidney, testis, placenta, gastrointestinal tract, and brain, all of which are, therefore, potential targets for SARS‐CoV‐2 infection.^30^ This extensive target disposition provides the opportunity for very broad‐based human cellular tropism.

### 4.2 The molecular basis for SARS‐CoV‐2 tropism

The molecular basis for understanding SARS‐CoV‐2 tropism is fundamental in predicting what cells in the human are prone to infection and this information informs the pathophysiology of COVID‐19 disease. It is also highly likely that SARS‐CoV‐2 tropism largely determines the nature of the progression of COVID‐19 in the human. This follows from the observation that SARS‐CoV‐2 infection is associated with early nasopharyngeal viral shedding raising the possibility of a tropism to the throat with this virus.^34^

Much has been written on the structural biological characteristics of the interaction of the SARS‐CoV‐2 spike glycoprotein with hACE2 and the focus here is briefly on the molecular features of this interaction that underpin SARS‐CoV‐2 tropism.

SARS‐CoV‐2 appears well suited to binding to hACE2 in that five amino acid changes on the SARS‐CoV‐2 spike glycoprotein are associated with the natural selection for critical binding sites (L455, F486, Q493, S494, N501) and responsible for the high tropism with hACE2.^28^ However, it is becoming apparent that the precise nature of the interaction of the amino acid residues on the SARS‐CoV‐2 spike protein depends on its configuration. There is accumulating evidence suggesting that the RBD of SARS‐CoV‐2 can oscillate between an “up” or a “down” configuration.^30^, ^35^ In the “down” position the RBD is associated with ineffective receptor binding, immune evasion^35^ and the interaction with ACE2 inhibited with stearic inhibition.^30^ In the “up” position greater than 16 amino acids in the RBD interact with hACE2.^30^ The change in RBD conformation between the open and closed states in SARS‐CoV‐2 occurs with exposure of the binding interface to ACE2, which causes the rotational motion of the whole RBD. Additionally, whereas SARS‐CoV and SARS‐CoV‐2 both have flexible regions in their RBD, SARS‐CoV‐2 also has flexible regions within the binding interface, which favor or disfavor binding.^29^

It is important to recognize that SARS‐CoV‐2 infection is dependent not only on the virus binding to ACE2 but the assistance of the pro‐protein convertase furin in the polybasic cleavage at the junction of S1 and S2. This allows the subsequent cleavage of the S2 site by TMPRSS2, which exposes the internal fusion motif peptide that is required for membrane fusion.^30^ This pre‐activation of SARS‐CoV‐2, unlike that of SARS‐CoV reduces its dependence on target cell proteases for entry.^5^ Although the efficiency of furin in SARS‐CoV‐2 S protein cleavage may be a distinguishing feature of SARS‐CoV‐2 in being more aggressive than other coronaviruses,^30^ the TMPRSS2 proteins have high homology across hosts and therefore would not appear to be involved in host selectivity.^36^ However, an important throat tropism seen with SARS‐CoV‐2 and not SARS‐CoV may be due to the presence of the polybasic furin cleavage site at the S1/S2 junction present in the SARS‐CoV‐2 virus.^34^

It follows that when viewing SARS‐CoV‐2 infection within the human, the affinity and high suitability for ACE2 binding on cells and tissues together with the furin mediated efficiency are the fundamental determinants of tropism. The co‐location of TMPRSS2 is a necessary attending requirement for this tropism to be functional. The amino acids involved in these protein interactions determine the thermodynamic potentials.^7^

### 4.3 Thermodynamic spontaneity and SARS‐CoV‐2 tropism

The association of the virus ligand with the ACE2 receptor is determined by the extent of the negative GFE value and this in turn determines the stability of the complex or the binding affinity of the ligand. Using homology modelling based on atomic details, Sakkiah et al. determined the interactions between the trimeric spike protein and ACE2 and demonstrated that the spike protein binds tightly with ACE2 with an estimated binding free energy of −60.54 kcal/mol.^37^ It follows that the properties associated with SARS‐CoV‐2 tropism are associated with a negative GFE value for the binding of this virus to its ACE2 receptor.

Comparison of SARS‐CoV‐2 with other coronaviruses that infect humans is insightful regarding the free energy of binding of this virus to the ACE2 target. Although at least seven human coronaviruses have been identified, three (HCoV‐NL63, SARS‐CoV, and SARS‐CoV‐2) display binding to ACE2. It is noteworthy that the spike protein binding region of HCoV‐NL63 has a low sequence identity with SARS‐CoV and SARS‐CoV‐2.^29^ Of importance, SARS‐CoV and SARS‐CoV‐2 displayed a high sequence identity of about 73% using multiple sequence alignment analysis.^29^ The difference in binding affinity between SARS‐CoV and SARS‐CoV‐2 offers important insights into the thermodynamic spontaneity and SARS‐CoV‐2 tropism. For example, He et al. have reported that the binding free energy of the SARS‐CoV‐2 RBD‐ACE2 interaction is −50.43 kcal/mol, which is lower than that of the SARS‐CoV RBD‐ACE2 interaction (−36.75 kcal/mol), consistent with the higher binding affinity of SARS‐CoV‐2 for ACE2.^38^ In addition, the binding free energy contributions indicate that this higher binding affinity is due to the solvation energy contribution.

Shang et al. indicated that the SARS‐CoV‐2 RBD has a higher binding affinity to hACE2 than the SARS‐CoV RBD and these differences are related to structural features that change the type of bonds between the RBD and ACE2.^5^, ^7^ As described earlier, the RBD of SARS‐CoV‐2 can oscillate between an “up” or a “down” configuration and the less exposed entire SARS‐CoV‐2 spike has comparable or lower binding than SARS‐CoV due to this lower exposure. It follows that the significant free binding energy of SARS‐CoV‐2 is related to these conformational dynamics. Overcoming the energy barrier associated with this conformational change would be expected to facilitate the binding of SARS‐CoV‐2 to ACE2 and thereby subsequent entry into the host cell.^7^

In summary, although knowledge of prevailing viral and host tropism is extremely important, it becomes very powerful when combined with the measures of receptor kinetics. It is this combination of cellular and host tropism, thermodynamics and kinetics that drives the temporal and spatial characteristics of COVID‐19.

## 5 THE MIGRATION OF SARS‐CoV‐2 TO VECTORS AND TO THE HUMAN

### 5.1 The collective faciliatory roles of a common conserved viral target, viral tropism, inflammation linked infectivity, and thermodynamic spontaneity

As discussed, SARS‐CoV‐2 cellular and host tropism, thermodynamics and kinetics are the fundamentals that underpin COVID‐19 disease. However, alone they are insufficient to drive the passage of SARS‐CoV‐2 from the host reservoir to the human.

In viewing the efficient passage of a virus from one species to another, the following three considerations deserve attention. There must exist a shared highly conserved viral binding target between the host reservoir and the new host. Within a new host, the physiological response should ideally support further spread and infectivity within the species. Thirdly, the binding affinities of the virus to the conserved sites within the new host must be consistent with the principles of thermodynamic spontaneity and mass action.

Focus has been drawn to bat species by virtue of their ability to accommodate many viruses including zoonotic coronaviruses and to harbor more zoonotic pathogens than any other known mammalian species.^39^ Bats can transmit viruses within‐host with minimal pathology^40^ and can display ACE2 receptor viral binding.^41^ Recent emerging viral disease outbreaks including Hendra, Nipah, Marburg, Ebola, SARS, and MERS have been linked to bat‐borne viruses.^39^

Although it is believed that SARS‐CoV‐2 may infect bats, direct evidence has been lacking and the molecular basis is still not fully understood.^42^ It is important to consider that there are approximately 1400 species of bats and, as Yan et al. demonstrated, ACE2 receptor usage in bats illustrates variation in the susceptibility to SARS‐CoV and SARS‐CoV‐2 infection among bat species.^41^ Moreover, there is at least a 79.6% shared genome sequence identity between the two viruses as well as ACE2.^43^ As highlighted by Irving et al., SARS‐CoV‐2 is thought to have ancestral origins in bats.^39^

Finally, understanding the relationships between hosts and viruses in bats provides the potential to gain important insights into the mechanisms used to avoid the pathology from virulent pathogens.

### 5.2 Passage of SARS‐CoV‐2 from vectors. Conserved host viral binding site combined with high rates of mutation and recombination

The passage of SARS‐CoV‐2 from one species to another is the genesis for the spatial development of COVID‐19 disease in humans. Woolhouse et al. showed that a potential determinant of whether a virus will transit from one species to another is its ability to use a common receptor which is conserved across both species.^44^ The ability of the virus to recognize the host binding site dictates the preference of the virus for both species and tissues.^28^ The high rates of mutation and recombination lead to variability in RNA viruses consistent with a more frequent jump between species than with other pathogens.

As mentioned earlier, the initiating event with SARS‐CoV‐2 viral infection involves the binding of the SARS‐CoV‐2 spike protein with ACE2. In 2010 Yu et al. highlighted that ACE2 proteins is highly conserved across mammalian species and a group of key amino acid residues are associated with the susceptibility of ACE2 to SARS‐CoV infection.^45^ Consistent with the predeterminants outlined by Woolhouse et al.,^44^ methodical mapping of ACE2 orthologs by Liu et al. across a wide range of species showed that SARS‐CoV‐2 has a broad host range at the level of viral entry that could contribute to cross‐species transmission.^42^ In a similar fashion, Damas et al. examined data sets from 410 vertebrate species, including 2252 mammals, to examine the cross‐species conservation of known binding residues of ACE2 and viral binding propensity.^46^ An extremely broad host range for SARS‐CoV‐2 may be a consequence of the conservation of ACE2 in mammals.^46^ Damas et al. noted that only mammals were defined by the medium to very high categories and that vertebrate classes other than mammals are not likely to be hosts for the virus.^46^ Furthermore, in exploring the evolution of ACE2 variation in vertebrates, it was concluded that the major ACE2 codons are significantly conserved, possibly reflecting the critical function of the renin–angiotensin system.^46^ Moreover, they observed that 10 residues in the ACE2 binding domain are exceptionally conserved in the Chiroptera (bat) family.

Based on an analysis of 70 ACE2 placental orthologues and using 30 critical ACE2 binding sites, Fam et al. concluded that there existed a high diversity of ACE2 between mammalian species.^28^ The broad host range in mammals is also dramatically illustrated in the bat. In contrast to a single human species, there are approximately 1400 species of bat, with ACE2 receptor viral usage, that is species dependent.^47^ Fam et al.’s comprehensive study of binding and infection assays examined 46 ACE2 orthologues from phylogenetically diverse bat species found that even closely related bat species showed different ACE2 proteins with some failing to support infection by either SARS‐CoV or SARS‐CoV‐2.

Human variation in ACE2 in the population is rare and intolerant to loss of function mutations.^46^ This is in stark contrast to other mammals that display a broad host range of ACE2 binding propensity for SARS‐CoV‐2 as discussed above. Importantly the studies of Fam et al. show a high diversity of ACE2 between placental mammals with no polymorphisms within the human population as measured at interspecies variable sites.^28^ *H*. *sapiens is* a highly populated single species with a static and conserved viral entry receptor. By way of summary, ACE2 is a highly conserved protein in mammals displaying an extremely broad host range for SARS‐CoV‐2 binding. In contradistinction, humans as a single mammalian species have constrained ACE2 variability, exhibit a high binding affinity for the virus and have the potential to be a recipient in the viral species jump driven in large part by a highly conserved protein target (Figure 4).

### 5.3 Passage of SARS‐CoV‐2 from vectors—The inflammasome and infectivity

The inflammatory response plays a key role in pathogen‐driven infective disease. Inflammation can serve as a protective response on the one hand but if dysregulated can act to enhance pathophysiology. Thus, host regulation of the physiological consequences of viral infection is an important determinant in viral infection and spread. The view that the bat is believed to host more zoonotic pathogens than any other known mammalian species may be a consequence, in part, of the bats’ ability to regulate host infection to prevent excessive immune‐driven pathology and a failure to display the clinical signs of inflammatory‐based disease when infected by viruses.^39^ Intriguingly, bats may limit viral load with the antiviral cytokine interferon‐alpha (IFN‐α) which predictably in mammals would normally be associated with inflammation; however, in bat adaption has apparently curtailed this inflammatory response. In a key study using viral dynamics, Brook et al. explored the transmission rates of IFN‐mediated immunity in bat cell lines that displayed either a constitutive or induced IFN response.^40^ They demonstrated that cells were protected from mortality with the antiviral state induced by the IFN pathway and suggested that the enhanced IFN capabilities achieve a more rapid within‐host transmission rate without pathology. Similarly, mammalian cells will induce the secretion of type I IFN proteins (IFN‐α and IFN‐β), which affect the expression of interferon‐stimulated genes (ISGs) in bystander cells, promoting an antiviral response and a predicted harmful immune inflammation.^40^ By way of summary, the bat may limit its viral load by way of antiviral cytokines and concurrently regulate the predicted inflammatory response through adaption.

As highlighted by Xiao et al., a natural reservoir host does not display severe disease.^48^ In contrast, an intermediate host may exhibit aspects of clinical infection. In this context, it is noteworthy that Xiao et al. reported that SARS‐CoV produced pathological changes, including interstitial pneumonia and diffuse alveolar damage in civets, like those observed in human SARS patients and those described for SARS‐CoV‐infected macaques.^49^ Similarly, pangolins shown to be positive for beta coronavirus showed clinical signs of disease, that is, diffuse alveolar damage of varying severity in the lung.^48^

The contrasting responses to viral infection both within bats to IFN‐α or in infected putative intermediate hosts outlined above raise several critical questions. Firstly, what are the key mechanisms to prevent excessive immune‐driven pathology in species that are potential asymptomatic viral reservoirs? Secondly, do viruses that maintain rapid replication in an environment of active immune defense potentially pose a threat to hosts not possessing similar immune properties of the bat^40^?

It has been suggested that the key to the regulation of the immune response in the bat is the inflammasome sensor NLRP3 (NOD‐like receptor [NLR] family pyrin domain‐containing 3). The NLRP3 inflammasome is involved in virus‐associated illnesses. Yap et al. have highlighted the importance of the inflammasome pathway in COVID‐19.^50^

Of particular significance is the demonstration that the NLRP3 inflammasome is dampened in bat primary immune cells compared with those of human or mouse, and this dampened response does not affect viral load.^51^ As indicated by Lara et al. this attenuated inflammatory response was also evident after infection by zoonotic RNA viruses such as influenza A, PRV3M, and MERS.^52^ Anderson et al. suggested that the dampening is consistent with the unique asymptomatic characteristics of bats as a viral reservoir.^51^

Not surprisingly, as a mammal, humans also use anti‐viral IFNs and have the capacity for inflammasome assembly and the formation of proinflammatory cytokines by way of caspase activation. As will be seen later, there is an important distinction between initial upper respiratory tract infection and serious COVID‐19 disease with lower respiratory infection in the human; upper respiratory infection is associated with a suppression of the type I IFN proteins (IFN‐α and IFN‐β), which dampens the expression of the proinflammatory ISGs. In contrast, lower respiratory serious COVID‐19 disease is associated with severe cytokine‐driven inflammation.

Since a lower respiratory cytokine storm is a hallmark of serious COVID‐19 disease, it is not surprising that focus has centered upon the NLRP3 inflammasome response with SARS‐CoV‐2 infection.^18^, ^50^, ^52^ Lara et al. have proposed that the NLRP3 inflammasome is key in the lethality in elderly patients with COVID‐19.^52^ They propose that aged individuals show a constitutive increase in pro‐inflammatory cytokines induced by NLRP3 activation of the hyper‐inflammatory cascade.

There is general agreement that in serious COVID‐19 disease, dysregulation of the angiotensin (Ang) II/AT_1_R (Ang II type 1 receptor) and Ang‐(1‐7) axis precipitates a hyper‐inflammatory state. There are possible links between the angiotensin‐based dysfunction and the NLRP3 inflammasome response in SARS‐CoV‐2. The role of SARS‐CoV‐2 in interacting with ACE2 and escalating the host response to infection into a dysregulated uncontrolled inflammatory response has been highlighted recently.^53^

In pulmonary tissue, Ang II binds to the AT_1_R and activates the NLRP3 inflammasome through intermediates.^54^ Hyperactivation of AT_1_R receptors by Ang II can produce excessive activation of the NLRP3 inflammasome and pyroptosis in a variety of cell lines including lung epithelium.^55^

In pulmonary tissue, Ang II is metabolized to Ang‐(1‐7). In lung fibroblasts, Ang‐(1‐7) has an inhibitory effect on the Ang II‐induced NLRP3 inflammasome through intermediates.^54^ Furthermore, in endothelial cell studies, Romero et al. have demonstrated that the vascular protective actions of Ang‐(1‐7) include the Nrf2 system.^56^ Ratajczak et al. demonstrated in human cells that Ang II ameliorated the activation of the NLRP3 inflammasome after interaction with SARS‐CoV‐2 S protein.^55^ These features are summarized concisely by Zhang et al. and include a SARS‐CoV‐2‐mediated decrease in Ang‐(1‐7), weakening the inhibition of the NLRP3 inflammasome and concurrently Ang II by way of AT_1_R activating the NLRP3 inflammasome.^57^

In summary, it is the dampening of primary immune cells involving the inflammasome in asymptomatic viral reservoirs that is like the initial upper airway infection in COVID‐19 disease in the human and this contrasts vividly with the hyper inflammatory state induced in the human lower respiratory tract with SARS‐CoV‐2.

### 5.4 Broad genetic diversity in the ACE2 binding of SARS‐CoV‐2 in the bat

Damas et al., using an ACE2 sequence data set, compared the properties of 410 vertebrate species including 252 mammals using 25 amino acids important for the binding between ACE2 and the SARS‐CoV‐2 spike protein.^46^ On this scale, 37 bat species scored low or very low on the potential to be used as a receptor by SARS‐CoV‐2. In an earlier study, Hou et al. showed that the diversity of ACE2 among bats was greater than that observed in all known SARS‐CoV susceptible mammals and that this genetic diversity stands in contrast to the homogenous ACE2 in humans.^58^ This diversity of ACE2 among bats is evident in the binding of SARS‐CoV‐2 to ACE2 within *Rhinolophus* bats. Human ACE2 lack its active‐site histidines (hACE2‐NN‐CT).^59^

In particular, Mou et al. found SARS‐2‐RBD bound *Rhinolophus macrotis* and *Rhinolophus affinis* ACE2 orthologs but did not bind *Rhinolophus pusillus* or *Rhinolophus sinicus* ACE2, suggesting a high degree of selection pressure on ACE2 from the SARS‐CoV‐2 virus within the *Rhinolophus* genus.^59^ Binding and infection assays have been used to examine 46 ACE2 orthologues from phylogenetically diverse bat species to support infection by SARS‐CoV or SARS‐CoV‐2. The study confirmed that ACE2 is the species‐specific entry receptor for SARS‐CoV and SARS‐CoV‐2. Moreover, they demonstrated that many bat species, 32 and 28 of 46 species, did not support the efficient binding with SARS‐CoV‐RBD and SARS‐CoV‐2‐RBD, respectively,^41^ consistent with earlier findings showing that bat ACE2s were less efficient overall than the hACE2 in relation to the susceptibility of SARS‐CoV entry.^58^

This lower efficiency of bat ACE2 (bACE2) compared with hACE2 may also occur with SARS‐related CoVs (SARSr‐CoVs). As highlighted by Guo et al., the bat *R*. *sinicus* carries SARSr‐CoVs, and they demonstrated that the SARSr‐CoV spike proteins had a higher binding affinity to hACE2 than to bACE2.^60^ They provided additional evidence to suggest that SARSr‐CoV co‐evolved with the host *R*. *sinicus* for a long time.^60^ It also suggests the possibility of infection into humans is, therefore, possible.

### 5.5 A potential affinity binding gradient between the natural reservoir of SARS‐CoV‐2 and the human

Previous experience with the single‐stranded RNA influenza A virus with its high mutation rate provides valuable insights into interspecies viral transmission. For example, the role of a change in viral tropism in interspecies transmission that occurs with a shift in receptor binding specificity of the influenza A virus is mutation determined.^27^ It has been the molecular insights into the haemagglutinin (HA) viral glycoproteins that bind to sialylated host cell receptors (and mediates membrane fusion) that have provided insights into the ability of several of the influenza A virus subtypes to jump from avian to human hosts.^27^ It is evident that the same broad principles relating to a shift in receptor binding characteristics and viral tropism with interspecies transmission may be applicable to SARS‐CoV‐2 infection.

**FIGURE 3:**
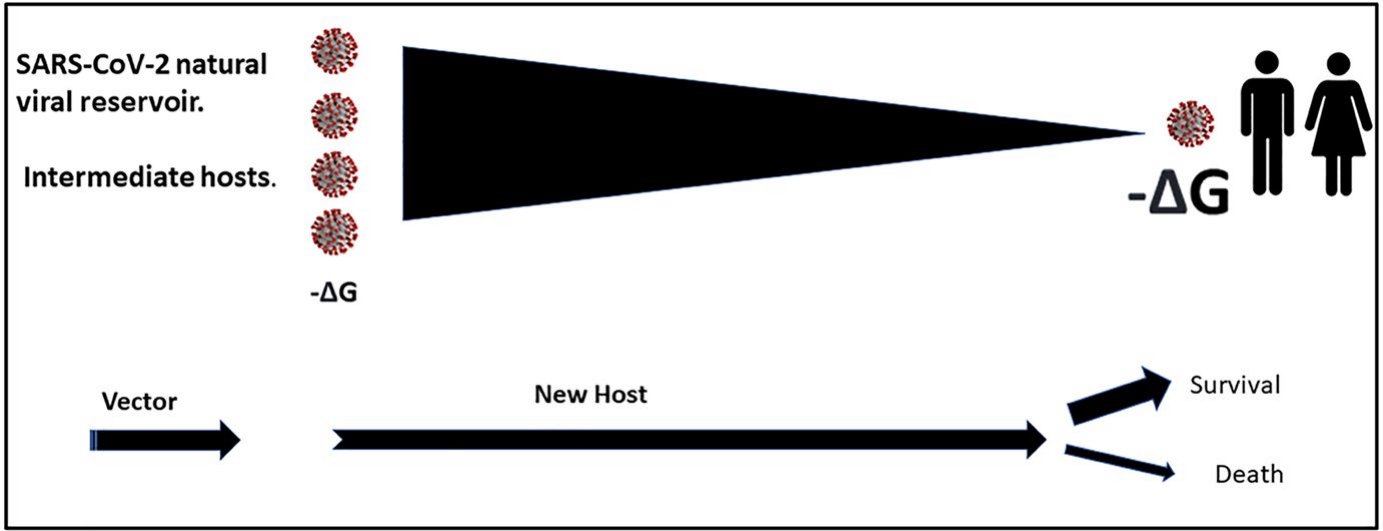
Gibbs free energy of binding and K_d_ (affinity constant) for viral binding of SARS‐CoV‐2 to ACE2 as potential faciliatory mediators of species jump. Illustration of a potential gradient underpinning the interspecies transmission for SARS‐CoV‐2 based on favorable thermodynamic spontaneity due to a higher affinity of the virus for hACE2. As indicated in the text, this pattern is not dissimilar to that described for influenza A, with a change in tropism and a shift in receptor binding specificity

A key study used the structural analysis of the interfaces of the SARS‐CoV RBD and host receptors to determine the principles that govern host adaptions and cross‐species infections and, importantly, the ability of SARS‐CoV to engage ACE2.^61^ Subsequently, Mou et al. demonstrated that SARS‐2‐RBD bound the *R*. *macrotis* ACE2 ortholog,^59^ and Liu et al. described the cross‐species recognition of SARS‐CoV‐2 to bACE2 and the RBD of SARS‐CoV‐2 that could bind to bACE2 from *R*. *macrotis*.^62^ Importantly, the binding mode of SARS‐CoV‐2 RBD and bACE2‐Rm was similar to the conserved binding mode of hACE2.^62^ This comparison is of significance in as much as it reveals that the SARS‐CoV‐2 RBD bound to bACE2‐Rm with much lower affinity (Kd = 0.44 µM) than to hACE2 (Kd = 20.4 nM). It could be anticipated then that the GFE for SARS‐CoV‐2 binding would be more negative with SARS‐CoV‐2/hACE2 interaction than for SARS‐CoV‐2/bACE2. This raises the possibility that a gradient in affinity exists between a natural reservoir of SARS‐CoV‐2 and the human, a gradient driven by viral tropism (Figure 3).

### 5.6 Potential intermediate hosts and affinity binding gradients

The coronaviruses SARS‐CoV‐1 and MERS are thought to have used palm civets (*Paguma larvata*) and dromedary camels (*Camelus dromedarius*), respectively, as intermediary hosts^63^ and are thought to have played a key role in transmission to humans.^64^ The possibility of a similar bridge host from natural reservoir host to humans is open to speculation; however, with regard to the importance of a cross‐species affinity gradient it deserves focus. It has been suggested that horseshoe bats (*R*. *affinis*) seem to be natural reservoir hosts.^65^ Li et al. demonstrated the binding of *R*. *affinis* ACE2 to the RBD, like that discussed above for *R*. *macrotis*, is markedly weaker than that of hACE2.^66^

Importantly Li et al. demonstrated strong binding to SARS‐CoV‐2 to Pangolin ACE2.^66^ Pangolins (*Manis javanica*) were considered as a primary suspect as a host bridge for SARS‐CoV‐2^64^ and appear to be an intermediate host for this virus.^65^ Although the role of pangolins as an intermediate host remains an area of discussion, it does illustrate the possibility that the cross‐species gradient affinity involves an intermediate host. This follows from the observation that the ability of the SARS‐2‐RBD to bind pangolin ACE2 was considerably weaker than the binding of SARS‐CoV‐2 to the monomeric forms of the tagged soluble hACE2 (hACE‐CT), and hACE2 lacking its active‐site histidines (hACE2‐NN‐CT).^59^ This suggests the viral binding to the putative natural viral reservoir ACE2 as well as the binding to a presumed bridging host ACE2 are both weaker than the binding to hACE2. This raises the possibility that a gradient in SARS‐CoV‐2 and ACE2 binding affinity exists between the natural reservoir as well as a bridging intermediate for SARS‐CoV‐2.

The studies highlighted above point to the existence of a very significant variation in susceptibility to SARS‐CoV and SARS‐CoV‐2 by way of binding to the ACE2 receptor. The available evidence thus points to the possibility of a higher affinity of SARS‐CoV‐2 binding to hACE2 than the equivalent binding in a natural reservoir, suggesting that the interspecies transmission for SARS‐CoV‐2 adheres to a pattern similar to that described for the influenza A virus, namely a change in tropism occurring with a shift in receptor‐binding specificity. It is this potential gradient in affinity of binding that may provide the thermodynamic spontaneity for the passage of SARS‐CoV‐2 from the natural reservoir to the human in COVID‐19 disease (Figure 3).

### 5.7 Compounding complex features of SARS‐CoV‐2 and ACE2 interaction between the natural reservoir of SARS‐CoV‐2 and the human

This discussion highlights the importance of the thermodynamic changes that occur with SARS‐CoV‐2 spike protein binding to hACE2 as an initiating or triggering event in infectivity associated with COVID‐19. However, as well as thermodynamics, there may be other complexities coming to light that explain the pivotal role of the RBD‐ACE2 interaction, including, at least in bats, the fact that SARS‐CoV may use an alternative receptor to ACE2.^67^ Furthermore, it is possible that not all of the transmission efficiency of SARS‐CoV‐2 is due to the affinity of the binding. Bats may need an intermediate host to amplify and spill over (such as in Hendra and Nipah infections). But the recent discovery of a bat SARSr‐CoV with an almost identical RBD of SARS‐CoV‐2 may suggest that, like Hendra and Nipah viruses there are bat SARSr‐CoV ready to jump to human. Lastly, the key RBD‐hACE2 interacting residues for SARS‐CoV‐2 are “flexible” in other RBDs.^67^ None of these residues are conserved in SARS‐CoV‐1 RBD, and yet both viruses spilled over into humans and caused major infection/diseases.

These considerations, although not necessarily eliminating a role of thermodynamic spontaneity for the passage of SARS‐CoV‐2 from the natural reservoir to the human in COVID‐19, suggest a degree of caution in basing an exclusive view that transmission is linked to the high affinity of SARS‐CoV‐2 for hACE2. A greater complexity may exist.

## 6 THE SARS‐CoV‐2‐INFECTED HUMAN

### 6.1 The nasal epithelium as the entry point for infection and transmission

The relentless drive underpinning the infection of a single cell, the passage from reservoir vectors to a new host, the success of viral variants and the infectious spread globally in a pandemic is a thermodynamics‐based complex system. The clinical properties of COVID‐19 display a time‐based evolution with three following time‐based phases. (i) The initial 1 to 2 days of infection with an asymptomatic state, (ii) followed by an upper airway and then a lower airway response, and (iii) progression for those with serious illness to acute respiratory distress syndrome (ARDS) and multiorgan failure.^68^ The evolution in a complex system has been described previously as time‐based thermodynamic evolution.^2^

The major mechanism of SARS‐CoV‐2 transmission in humans is by way of infected respiratory droplets and/or aerosols with nasopharyngeal viral shedding very early in COVID‐19 disease. Santos et al. drew attention to the observation that nasal swabs from patients with COVID‐19 display higher viral loads than do throat swabs, inferring a potential role of the nasal epithelium as an entry point for infection and transmission.^69^ Carcaterra and Caruso have also put forward a model highlighting that overcoming the first line of nasal defense and upper airway colonization is a key step for SARS‐CoV‐2.^70^ In a similar fashion, Bourgonje et al. indicated that SARS‐CoV‐2 may pass through the mucous membranes associated with nasal epithelia to bind to its target, ACE2.^71^ The entry point for infection provides the stage for SARS‐CoV‐2 to exert this powerful viral tropism and is also a key place for the development of pharmacological therapy development.

The following discussion explores the events associated with the initial infection in the upper respiratory tract. It is the fundamental role of nasopharyngeal tropism associated with SARS‐CoV‐2 in COVID‐19 that orchestrates the progression, direction, and severity of this disease via the diffusional viral gradients.

### 6.2 The critical role of nasopharyngeal SARS‐CoV‐2 tropism

One of the remarkable distinguishing features between SARS‐CoV and SARS‐CoV‐2 is the degree to which they infect the upper respiratory airways in the human. SARS‐CoV‐2 is more efficient in its transmission through pharyngeal viral shedding when symptoms are mild.^34^ In serious COVID‐19 disease, it can subsequently resemble the pathophysiology of SARS‐CoV in the lower respiratory tract. It follows that a major aspect of COVID‐19 is the high level of SARS‐CoV‐2 shedding in the upper respiratory tract compared with SARS‐CoV, where replication is mainly in the lower respiratory tract.^72^ This difference in the efficiency of transmission for SARS‐CoV and SARS‐CoV‐2 is borne out in the infectivity of the two viruses. Within eight months of emergence, SARS‐CoV was controlled and had infected approximately 8,100 persons. In contrast, after its emergence, SARS‐CoV‐2 had infected 2.6 million people.^73^ In addition, the peak SARS‐CoV‐2 viral load occurs within the first week of symptom onset, and this contrasts to SARS‐CoV where the peak is between 7 and 10 days of illness.^72^ Based on the high homology in structure between SARS‐CoV and SARS‐CoV‐2 and their dramatic differences in morbidity and mortality, understanding and contrasting the fundamental infective properties of these viruses will cast light on the role of SARS‐CoV‐2 in COVID‐19 disease.

Even though there is at least a 79.6% shared genome sequence identity^43^ and a common mammalian viral target receptor, ACE2, for SARS‐CoV‐2 and SARS‐CoV, there is a tropism exhibited with SARS‐CoV‐2 and not for SARS‐CoV for the upper respiratory tract. As summarized by Aguirre Garcia et al., although there can be similar replication rates between SARS‐CoV‐2 and SARS‐CoV in the lower respiratory tract, SARS‐CoV‐2 replicates 100‐fold more efficiently in the upper respiratory tract.^74^ One hypothesis to explain this extension of tropism is the presence of a polybasic furin cleavage site at the S1–S2 junction within SARS‐CoV‐2 but not SARS‐CoV, with the potential that it may lead to a gain‐of‐fusion.^34^ Earlier, we highlighted that the SARS‐CoV‐2 RBD can exist in an “up” or “down” configuration. The balance between having high infectivity as well as limiting the immune accessibility of the SARS‐CoV‐2 RBD is achieved by using host protease activation.^75^ As summarized by Aguirre Garcia et al., the S protein of SARS‐CoV‐2 is normally in a conformation evading immune interaction and with pre‐activation of the binding domain by furin enabling binding of SARS‐CoV‐2 to ACE2.^74^

A tropism of this type is not restricted to SARS‐CoV‐2, for similar features are seen with the influenza virus. In particular, avian influenza H5N1 or H7N9 viruses bind to 2,3‐linked sialic acid in the lung alveoli, causing severe pneumonia, whereas the seasonal influenza H1N1 and H3N2 bind to the α‐2,6‐linked sialic acid receptors of the upper respiratory facilitating more transmission.^76^ In current highly pathogenic avian influenza viruses, the increased virulence in mammalian hosts is also associated with the presence of a multibasic cleavage site.^77^

By way of summary, there is a different viral tropism for SARS‐CoV‐2 and SARS‐CoV in the respiratory tract resulting in a highly transmissible disease when SARS‐CoV‐2 replicates in the upper respiratory tract.^76^ For both viruses, less transmission and the risk of severe pneumonia are associated with lower respiratory tract replication. It is unlikely that the viral tropism for SARS‐CoV‐2 displayed for the upper respiratory tract is due to the gain in fusion mediated by the polybasic furin cleavage site acting in isolation, but rather in concert with the constellation of events associated with the colonization of the upper respiratory tract.

### 6.3 The role of binding affinities and mucosal diffusion in driving upper airway SARS‐CoV‐2 tropism

The first interaction between an individual and airborne SARS‐CoV‐2 is at the mucosal surface in the upper respiratory tract. This protective mucosal barrier is a major component of the nasal cavity and the nasopharyngeal‐associated lymphoid tissue (NALT).^78^ This mucosal barrier is an innate defensive system^68^ and the mucosal immune system is both the largest component of the immune system and is the point of first encounter of SARS‐CoV‐2 with the immune system.^79^ The nose and the NALT are key in the induction of mucosal immune responses including the generation of Th1‐and Th‐2 polarized lymphocytes and IgA committed B cells.^78^

For SARS‐CoV‐2 to gain access to the nasal epithelia to bind to ACE2, it must traverse two mucosal layers. The upper viscous layer sits above the less viscous periciliary layer (PCL) and the diffusion of molecules into this layer is hindered by the membrane spanning mucins and mucopolysaccharides associated with the cilia and epithelium.^80^ The viscous gel layer contains the secreted mucins (MUC5AC and MUC5B) and the PCL contains the membrane tethered mucins (MUC1, MUC4, and MUC16) providing a molecular brush. The mucins are heavily glycosylated with about 25 to 30 carbohydrate chains per 100 amino acids with complex glycan chains containing mainly O‐glycans. It is important to note that the SARS‐CoV‐2 spike protein is also heavily glycosylated by the glycosylation apparatus from a previous host as the virus passes through its secretory pathway.^32^ In essence, the passage of SARS‐CoV‐2 through the mucosal layers reflects that of a heavily glycosylated virus through an environment of heavily glycosylated mucins.

Lee et al. demonstrated that the ACE2 protein is abundantly expressed in multiciliated airway epithelial cells from the nasal cavity to the bronchus and not in the secretory goblet cells of the airway epithelium.^81^ Additionally, Sungnak et al. demonstrated that both ACE2 and the entry associated protease, TMPRSS2, are highly expressed in nasal ciliated cells and epithelial cells.^23^ Lee et al. proposed that the enrichment of ACE2 in the motile cilia of the nasal cavity would suggest a capacity for early subcellular site for SARS‐CoV‐2 entry.^81^ Consistent with this view, Chatterjee et al. suggested that nasal carriage is likely to be a feature of viral transmission.^68^

In a landmark study, Hou et al. examined viral tropism along the human respiratory tract and demonstrated a gradient in infectivity for SARS‐CoV‐2 from the proximal to the distal respiratory tract.^82^ Of significance was the observation that the amounts of ACE2 waned in the more distal bronchiolar and alveolar regions and that these expression patterns were paralleled by high SARS‐CoV‐2 infectivity in the nasal epithelium with a gradient reduction in the bronchioles and alveoli in the distal lung.

By way of summary, ACE2 (the target for SARS‐CoV‐2) are in the motile cilia of epithelial cells in the nose and the paranasal sinuses. These are localized adjacent to the tethered mucins within the PCL with a gradient in infectivity from the upper to lower respiratory tract. However, the extent to which SARS‐CoV‐2 can infect the multiciliated airway epithelial cells is determined by the effectiveness of an additional diffusion gradient, namely, a diffusional gradient spanning the mucosal barrier from the air interface to the underlying ACE2 enriched multiciliate airway epithelial cells.

The following processes will influence that diffusion:

### 6.4 The role of a mucosal diffusional gradient

The binding affinity of SARS‐CoV‐2 for ACE2 is viewed as one of the contributors to COVID‐19. As mentioned earlier, the binding free energy for the SARS‐CoV‐2 RBD‐ACE2 interaction is approximately 24 kcal/mol more negative than that for SARS‐CoV, a virus that displays minimal interaction with the upper respiratory tract.^38^

Moreover, the polybasic cleavage site at the junction of S1 and S2 present in SARS‐CoV‐2 and not SARS‐CoV, provides for efficient cell entry mediated by furin and TMPRSS2. In kinetic terms, one can anticipate that in the region of the nasal epithelium, the high affinity of SARS‐CoV‐2 for ACE2 and the efficiency of the extracellular removal of the virus is a powerful combination that in kinetic terms (high affinity and product removal) facilitates the passage of this virus across the mucosal layers. This combination is expressed as SARS‐CoV‐2 tropism for the upper respiratory tract.

As indicated earlier the diameter of SARS‐CoV‐2 is approximately 100 nm and the thickness of the mucus layer that covers the epithelial cells in the airways is in the range of 7 to 70 µm^83^ suggesting that SARS‐CoV‐2 has to traverse a barrier some 70 to 700 times its diameter to be within the region of the epithelial cell tethered ACE2 receptor.

**FIGURE 4:**
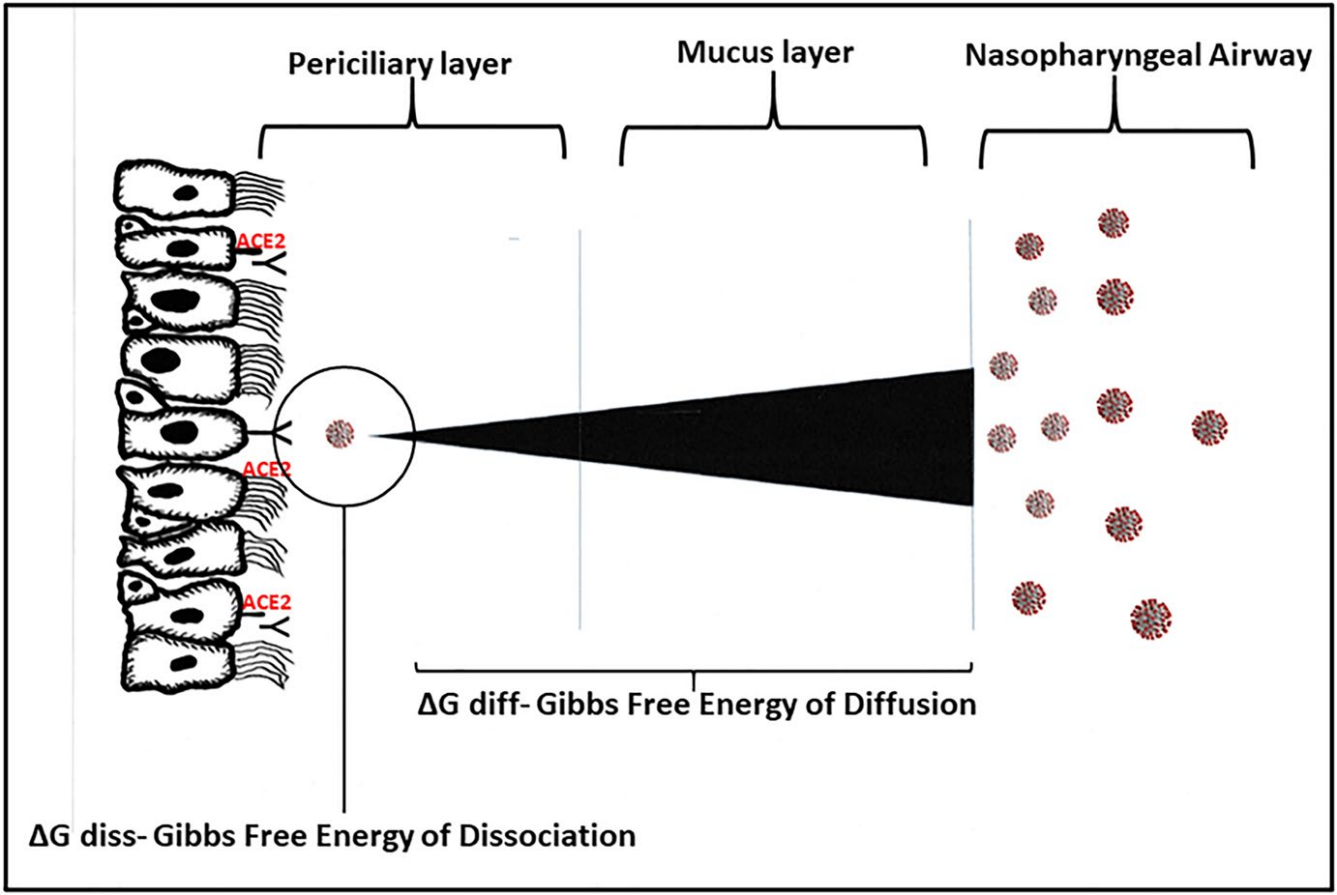
A schematic representation of a mucosal gradient for the passage of SARS‐CoV‐2 from the upper respiratory tract mucosal airway intersection to the epithelial cell–bound ACE2 target. The polybasic cleavage site at the junction of S1 and S2 present in SARS‐CoV‐2 likely provides for efficient cell entry and removal from the mucosal layer, thus ensuring that the concentration of the virus will be minimal compared with that which may be present at the mucosal airway surface. The figure also highlights the importance of Gibbs free energy in both the diffusion of SARS‐CoV‐2 across the mucosal layers and the subsequent interaction with its ACE2 receptor

If the passage of the virus is impeded in the mucosal layer and the diffusion of virus particles is not sufficient to achieve an equilibrium concentration across the mucus layers including the PCL adjacent to the epithelial bound ACE2 target, then a gradient will be established that is analogous to the well‐described pharmacological agonist concentration gradients (Figure 4). Of significance is that the nonequilibrium state implies that there is free energy available to do work and that this viral mucosal migration can be described by the GFE of diffusion.

It should be noted that the passage of a virus particle across the mucosal layers is not totally assured despite this high affinity and efficiency. This follows from the fact that mucins may serve as binding sites for pathogens and may, through steric hindrance, also modulate the binding of a virus to its epithelial cell bound receptor.^84^ In this context, understanding the nature of glycosylation of the virus and the mucins is fundamental.

As mentioned earlier, the spike protein of SARS‐CoV‐2 and the mucosal layers through which the virus must traverse are both heavily glycosylated. The following glycan considerations are of importance in relation to the diffusion of SARS‐CoV‐2 across the upper airway mucosal barrier.

As summarized by Hernández et al., based on cryo‐EM structures, the SARS‐CoV‐2 spike protein is highly glycosylated with a similar pattern to that of the SARS‐CoV‐1 spike protein, where of the twenty two N‐linked glycosylation sequons per promoter, 20 of these sequons of SARS‐CoV‐2 S are conserved in SARS‐CoV‐1 S.^85^ Likewise, the S2 subunit N‐linked glycosylation is also conserved.^85^ In contrast to bacteria where the glycans are encoded by the bacterial genome, the glycosylation of SARS‐CoV‐2 is the product of a previous mammalian host's cellular glycosylation processes. As summarized by Zhao et al., such bacterial glycans are viewed as “nonself” glycans. This glycan shielding for SARS‐CoV‐1S and SARS‐CoV‐2S may serve the dual purpose of avoiding immune sequestration and reduce the likelihood of impeded passage across the mucosal layers due to mucin binding of a nonshielded pathogen.^32^

At the epithelial surface, glycans may play an additional role in the docking of SARS‐CoV‐2 with ACE2. The ACE2 target has been reported to have seven N‐glycosylation sites and several O‐glycosylation sites and importantly, the glycan at N322 interacts tightly with the bound spike protein.^86^ Collectively, these suggest a role for glycosylation in the binding of SARS‐CoV‐2 to its receptor.

It is the SARS‐CoV‐2/ACE2 binding efficiency that determines SARS‐CoV‐2 transmissibility.^85^ SARS‐CoV‐2 but not SARS‐CoV‐1 infection is directed toward the upper respiratory tract. Based on the similarity of glycan shielding for both viruses, it seems unlikely that this difference in infectivity is due to differences in the ability of both viruses to diffuse across the mucosal layers. It seems rather more likely to be due to the 10‐to‐20‐fold higher target binding affinity for SARS‐CoV‐2 for the ACE2 receptor.^85^ It is this higher binding affinity coupled with the efficient furin facilitated cellular fusion and the removal of the virus at the surface of the epithelium that drives the glycan shielded SARS‐CoV‐2 to cross the mucosal layers. In this way, with exposure, the concentration of the virus particles will be minimal at the epithelial mucosal layer interface and higher within the mucosal bilayer.

The mucosal viral gradient driven by viral tropism and receptor affinity is the first critical event in infection of humans with SARS‐CoV‐2. It is the opportunity to enhance the efficiency of this process that is the primary objective of mutant variants of SARS‐CoV‐2 (Figure 4).

### 6.5 SARS‐CoV‐2 variants and mucosal diffusional gradient

Ou et al. examined the in silico binding interactions between SARS‐CoV‐2 mutants and hACE2 to determine the effect of naturally occurring RBD mutations on receptor binding affinity and infectivity.^87^ Of importance was the observation that the GFE (Δ*G*) of the V367F mutant was significantly lower (approximately 13 kJ/mol) than that of the original wild‐type strain. Moreover, the affinity constant (*K*_D_) of the wild‐type RBD was reported to be about two orders of magnitude higher than the *K*_D_ of the V367F mutant (14.7 and 0.11 nM, respectively) indicating an increased affinity of the mutant for hACE2. That is, compared with the *K*_D_ (14.7 nM) of the prototype RBD, the *K*_D_ of the V367F mutant was 0.11 nM, which is two orders of magnitude lower than for the prototype strain, indicating an increased affinity to hACE2. Subsequently, it has been established that mutations within the RBD focus on key sites (K417, L452, E484, N501) which enable the spike protein to avoid antibody neutralization and concurrently maintain or enhance binding to ACE2.^88^ A comprehensive description of the nature and significance of spike protein mutations as they relate to SARS‐CoV‐2 has been described in detail by Winger and Caspari, with relatively small changes in RBD having profound effects on viral infectivity.^88^

Evidence for increased infectious titers in nasal washes but not the lungs of hamsters infected with SARS‐CoV‐2 expressing spike D614G support the clinical data that these mutations enhanced viral loads in the upper respiratory tract of patients with COVID‐19.^89^ There is growing evidence that SARS‐CoV‐2 variants bind ACE2 with increased affinity, including a greater affinity of the B.1.1.7 (alpha) RBD‐bound ACE2, and the B.1.351 (beta) RBD‐bound ACE2. This likely contributes, in part, to the enhanced transmissibility.^26^

Recently, a highly transmissible Delta (b.1.617.2) variant has been documented that has seven spike protein mutations.^90^ So, in addition to the D614G mutation described earlier for opening the individual trimers in the SARS‐CoV‐2 trimers, the Delta variant has spike protein mutations that have a leucine to arginine substitution at position 452 on the RBD, which increases the affinity of this virus for ACE2.^91^ Concurrently in this mutant, there is a proline to arginine substitution at position 681 which renders furin more effective and primed for cell entry such that 10% of the spike proteins were primed in the original strain, 50% in the alpha strain and 75% in the Delta variant.^91^ During the course of the preparation and review of this manuscript a further highly transmissible variant, Omicron has been reported with substitutions T478K, Q493K, and Q498R contributing to the binding energies and a doubling of the electrostatic potential of its RBD and the ACE2 complex.^92^

The evidence for mutations enhancing the binding affinity for SARS‐CoV‐2 for the ACE2 receptor is compelling and provides a rational explanation for transmission at low viral loads and infectivity with current and emerging variants. The enhanced binding displayed by the variants lowers the concentration of virus particles required to complete the passage across the mucosal barrier and bind with epithelial cell–bound ACE2 and achieve successful infectivity. Additionally, there is increasing evidence that the SARS‐CoV‐2 variants of concern have higher viral loads, longer viral shedding time, and shorter incubation periods.^93^

**FIGURE 5:**
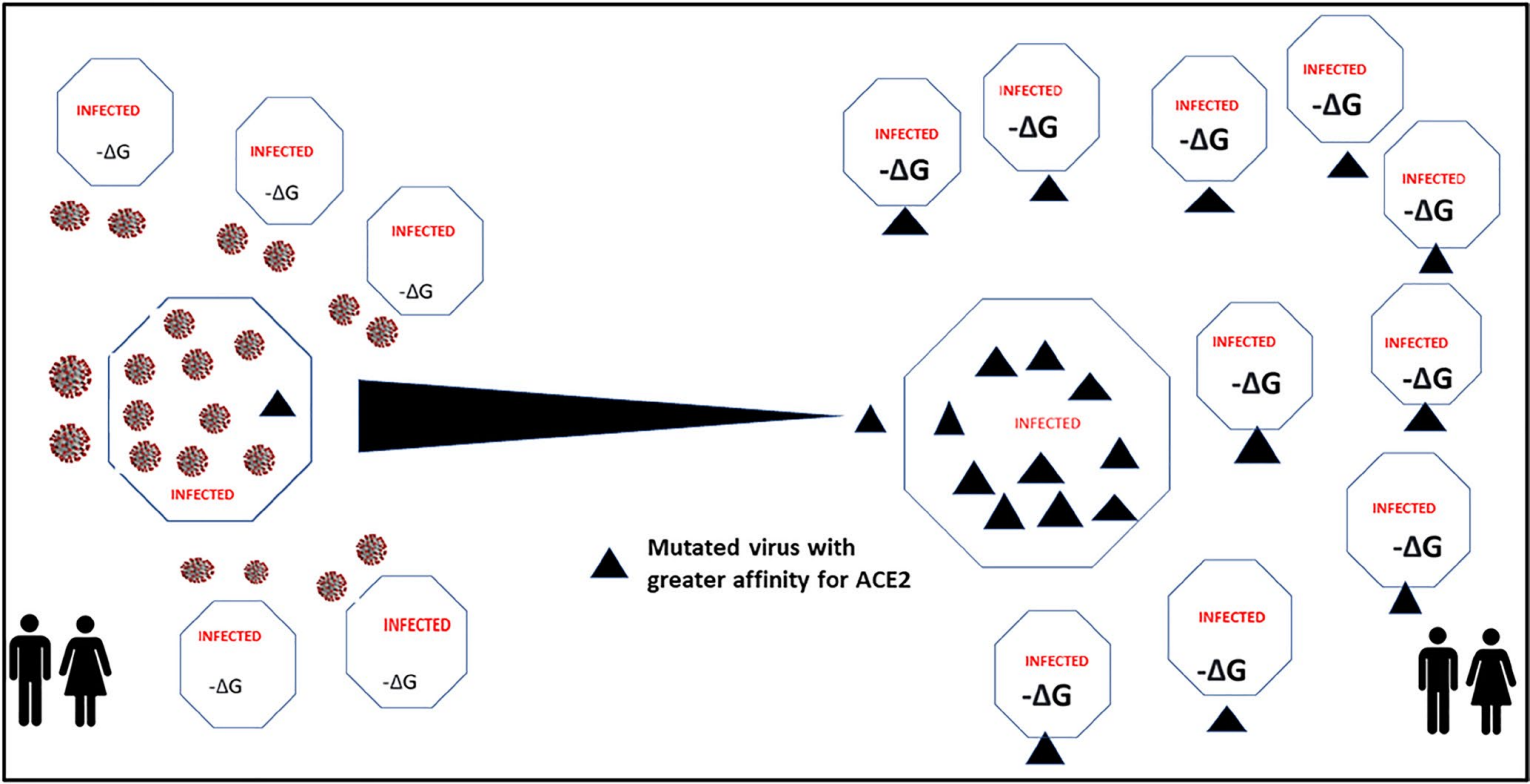
Gibbs free energy of binding and Kd (affinity constant) for enhanced ACE2 binding and infectivity of SARS‐CoV‐2 variants. A schematic representation illustrating how the greater affinity of SARS‐CoV‐2 variants lowers the concentration of virus particles required to complete the passage across the mucosal barrier and bind with epithelial cell–bound ACE2 and achieve successful infectivity. The schematic also portrays the efficiency of variant (▲) spread throughout bystander cells driven by repeated thermodynamic spontaneity as a consequence of the availability of Gibbs free energy (ΔG) associated with viral target binding. Wild type SARS‐CoV‐2 is schematically represented by the circular virus

It is the extent to which changes in GFE with variants favors the passage of SARS‐CoV‐2 and its variants across the mucosal gradient and the changing mutational‐driven efficiency in binding to the ACE2 receptor that is the bedrock for the initial dynamics and seriousness of COVID‐19 disease in humans (Figure 5).

## 7 HOW DOES NASAL SARS‐CoV‐2 INFECTION DICTATE MILD OR SERIOUS COVID‐19 DISEASE?

### 7.1 Asymptomatic responses with high viral loads

As highlighted above, COVID‐19 disease is the activation of a complex system that can be described by a time‐based thermodynamic evolution.^2^ The initial site for entry of SARS‐CoV‐2 is the mucosal surface. This is followed by diffusion across the mucosal bilayers, attachment to the ACE2 receptor, and entry within the nasal epithelium. The immediate events that follow upper airway infection dictate whether this disease is either mild or asymptomatic or alternatively progresses to serious disease.

What is generally accepted is the initial high viral loads in the upper respiratory tract suggesting a high viral RNA shedding potential for transmission^94^ and this pharyngeal shedding occurs at a time when symptoms are mild.^34^ What is at first consideration surprising is the absence of an early and significant nasopharyngeal inflammatory response. Moreover, it appears that in COVID‐19, a high number of asymptomatic patients exhibit positive nasopharyngeal viral detection, suggesting the lack of an immune response.^70^ These clinical observations are consistent with the lower expression of genes (TNF, IL32, IL1A, CXCL1, and CXCL3) associated with the acute inflammatory response to SARS‐CoV‐2 in lung epithelial cells.^95^

### 7.2 The dampened nasal interferon and inflammasome response

As summarized by Bridges et al., a dampened IFN response with a suppressed immune response to SARS‐CoV‐2 in the nasal passage complex is likely to be pivotal.^96^ Normally, viral regulation by foreign pattern recognition molecules occurs through a cascade of transcription factors and release of IFNs and cytokines. During SARS‐CoV‐2 replication, TLR‐3 receptors induce an immune response that results in the production of type I IFNs and proinflammatory cytokines and it is the expression of type I IFN that protects noninfected cells by releasing antiviral proteins.^85^ It is the release of type I and type III IFNs and other cytokines that stimulate IFN sensitive genes in both infected and noninfected bystander cells.^96^ Mammalian cells will induce the secretion of type I IFN proteins (IFN‐α and IFN‐β), which affect the expression of ISGs in bystander cells promoting an antiviral response and a predicted harmful immune inflammation.^40^ Brodin drew attention to the ability of SARS‐CoV‐2 to inhibit type I IFN responses to allow the virus to replicate and induce more tissue damage.^97^ Furthermore, in comparison with other respiratory viruses, Blanco‐Melo et al. demonstrated that despite viral replication, the host response to SARS‐CoV‐2 fails to launch a robust IFN and IFN‐3 response.^98^ As pointed out by Galani et al., when acting appropriately the IFN‐mediated responses should precede the pro‐inflammatory response optimizing host protection and minimizing collateral damage suggesting that the antiviral response is untuned.^99^ Choi and Shin have drawn attention to type I and III IFNs as major first‐line defenses against viruses acting using pattern recognition receptors (PRRs).^100^ As summarized by Walker et al., type I IFNs restrict the spread of viruses beyond their initial mucosal site of infection, whereas type III IFNs restrict virus infections within mucosal tissues and may induce less inflammation than type I IFNs.^101^ The critical role of the mucosa in viral defense in COVID‐19 disease is discussed later in this review. Importantly and as highlighted by Choi and Shin, many viruses have mechanisms to evade and suppress the antiviral functions of IFNs including the coronaviruses SARS‐CoV‐1 and MERS‐CoV that suppress PRR activation.^100^ Moreover, there is the possibility that SARS‐CoV‐2 is even more efficient than other CoVs in inhibiting IFN signaling and activity.^100^ As highlighted by Ziegler et al., compared with other common respiratory viruses SARS‐CoV‐2 elicits poor type I IFN‐mediated responses and severe COVID‐19 is characterized by a dramatically blunted IFN response.^102^ By way of summary, Sposito et al., have suggested that efficient initiation of IFN in the upper airways can lead to more rapid elimination of the virus and may limit viral spread to the lower airways.^103^ If the virus escapes immune control in the upper airways, the IFN production in the lungs likely contributes to the cytokine storm seen in patients with severe‐to‐critical COVID‐19.^103^

An alternative explanation for a dampened nasal response comes from kinetic studies in bat cells on the transmission rates of IFN‐mediated immunity, where it was suggested that it is possible to achieve a more rapid host transmission rate without the pathology.^40^ It is highly likely that this delayed inflammatory response prolongs viral replication^95^ and would explain why asymptomatic patients with COVID‐19 disease have significant viral loads and can infect others. Consistent with this view are the findings of Mick et al. that the IL‐1 and NLRP3 inflammasome pathways were nonresponsive to SARS‐CoV‐2 consistent with impaired neutrophil and macrophage recruitment, explaining high viral load prior to symptom onset.^104^ This nasal airway regulatory response is also entirely consistent with earlier studies with rhinovirus that demonstrated regional differences in epithelial airway cells and in particular, a trade‐off between viral defense and oxidative stress protection detailed by Mihaylova et al.^105^ These authors demonstrated that nasal cells display a predominately IFN response, whereas bronchial cells exhibit a predominant oxidative stress response.

The potential activation of the NLRP3 inflammasome in response to SARS‐CoV‐2 infection has been discussed in detail and it is noteworthy that one of the pathways includes the loss of a capacity to hydrolyze Ang II by virtue of the SARS‐CoV‐2/ACE2 interaction and, consequently, the activation of the NLRP3 inflammasome.^18^ It is likely that this NLRP3 mediated dampened response adheres to a broader response pattern for two reasons. Firstly, blunted activation of the IL‐1 and NLRP3 pathways is associated with an asymptomatic course of infection with human influenza challenge.^104^ Secondly, and of particular significance, is the demonstration that the NLRP3 inflammasome is dampened in bat primary immune cells compared with those of human or mouse and this dampened response does not affect viral load.^51^ In addition to the nasopharyngeal tropism outlined earlier, SARS‐CoV‐2 has used suppression of the predominately IFN‐driven upper airway response to enable greater viral titers with minimal inflammatory responses and greater asymptotic infectiveness in the human.

### 7.3 Progression to lower respiratory tract acute respiratory distress syndrome

It has been suggested that the virus migrates down the respiratory tract with the triggering of a more robust innate immune response and about 80% of those infected will have mild disease restricted to the upper respiratory tract and conducting airways.^106^ It could be argued that on viral shedding and luminal release of the virus that inhalation may lead to infection of alveolar cells by way of SARS‐CoV‐2 binding to ACE2. In a detailed analysis, Hou et al. drew attention to the importance of oral‐lung aspiration as a major contributor to many lower airway infectious diseases and the combination of muco‐ciliary clearance, accumulation of a bolus with viral titer in the oral cavity followed by aspiration to the lower lung.^82^

After progression to the lower respiratory tract, the SARS‐CoV‐2‐mediated targeting and impairment of ACE2 function is fundamental in the progression to serious disease in the lower respiratory tract for two reasons. Firstly, ACE2 is localized on the alveolar type II cells that are responsible for the production of surfactants, stabilization of the epithelial barrier, immune defense and regeneration following injury.^70^ With significant impairment of these cells by SARS‐CoV‐2 using ACE2 as the cellular target, the stage is set for the progression to ARDS. This progression has been well covered in the literature and involves the hyper‐inflammatory response associated with the “cytokine storm” associated with the initial exudative phase of ARDS. The critical observation is the contrasting dampened innate immune response in the upper airways associated with high viral loads and often with asymptomatic responses with the alveolar damage and hyperinflammatory immune response in serious COVID‐19 associated with the lower respiratory tract. Blanco‐Melo et al. have proposed that the reduced antiviral defense coupled with the enhanced cytokine inflammation are the driving and defining features of COVID‐19.^98^

**FIGURE 6:**
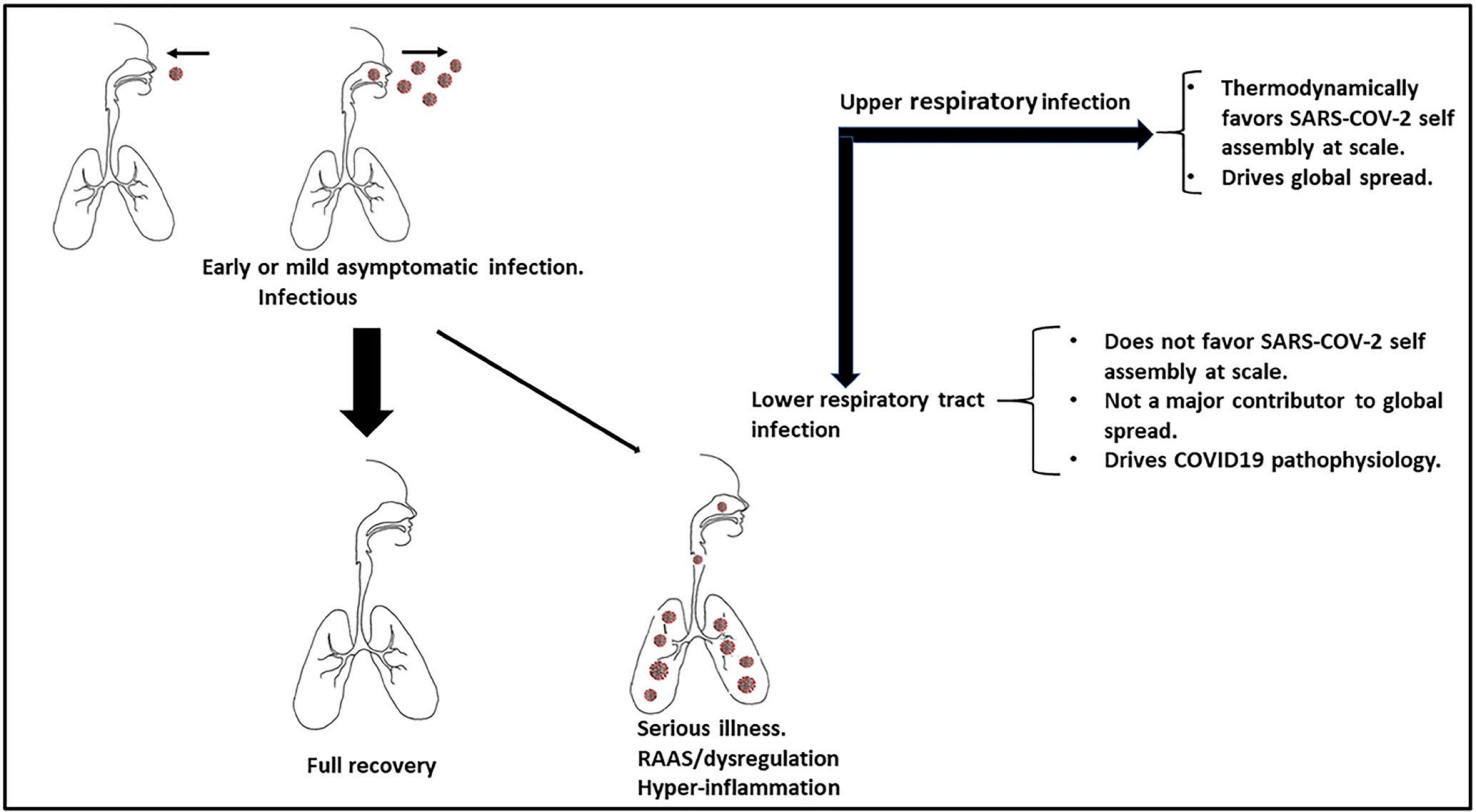
Schematic representation illustrating the progression of SARS‐CoV‐2‐mediated COVID‐19 disease from mild or asymptomatic infection to either full recovery or serious illness. Highlighted is viral self‐assembly at scale and population spread is mediated by upper respiratory tract infection. The fundamental pathophysiology of serious COVID‐19 disease is driven by lower respiratory tract infection and does not favor viral self‐assembly at scale

It is the presence or absence of this progression that dictates mild or serious COVID‐19 disease. This consideration provides a rational basis for why SARS‐CoV‐2 replicating early in the upper airways with minimal inflammatory response results in a more transmissible disease compared with SARS‐CoV‐1, and why for both viruses, less transmission and greater risk of severe pneumonia is the hallmark of lower respiratory tract replication (Figure 6).

From a thermodynamic standpoint, the highly infectious upper airway component of this disease, and its attending population spread, provides far greater changes in GFE than with the lower infectivity associated with the more serious lower respiratory COVID‐19 disease. This follows from the fact that the change in adsorption enthalpy reflects the sum of the bond energy changes, which will be far greater with higher levels of infectivity mediated by the upper respiratory tract.^7^

## 8 A UNIFYING THERMODYNAMIC PRINCIPLE FROM SINGLE CELLULAR INFECTION TO POPULATION SPREAD

The principal mechanism of SARS‐CoV‐2 infection is via respiratory droplets that contact nasal, conjunctival or oral mucosa.^107^ From the preceding discussions, it is apparent that SARS‐CoV‐2 has a higher affinity for ACE2, and the presence of the furin cleavage site imparts a high level of efficiency for entry of SARS‐CoV‐2 into cells containing the ACE2 receptor and predictably simultaneously reduce the concentration of the virus in the epithelial–mucosal interface. The latter can be viewed as the driver of the passage of SARS‐CoV‐2 across the mucosal barrier. Wolfel et al. drew attention to the potential extension of tropism to the throat with SARS‐CoV‐2 infection by way of the presence of the polybasic furin‐type cleavage site at the S1–S2 junction in the SARS‐CoV‐2 spike protein that is not present in SARS‐CoV.^34^ By way of summary, despite the high homology of SARS‐CoV‐2 and SARS‐CoV the furin site is not present in SARS‐CoV‐1 and the affinity of SARS‐CoV‐1 for ACE2 is not as great as seen for SARS‐CoV‐2.

A comparison of the characteristics of the initial host–viral interactions of the genetically similar SARS‐CoV‐1 and SARS‐CoV‐2 provides valuable insights into the marked infectivity of SARS‐CoV‐2 in COVID‐19 disease. This enhanced infectivity for SARS‐CoV‐2 is apparent with the higher reproductive number compared with SARS‐CoV‐1 highlighting a more efficient spread which is seen dramatically in the population‐based data for COVID‐19 disease.^107^

Additional differences also relate to fatality rate and these have been earlier summarized by Petrosillo et al., where estimates suggest a fatality rate of 2.3% for COVID‐19, 9.5% for SARS (mediated by SARS‐CoV) and 34.4% for MERS.^76^ As of September 2021, there have been 226.8 million confirmed cases of COVID‐19 disease with 4.6 million deaths (Source: World Health Organization. 14 September 2021) with a case fatality rate of less than 1% in 74 out of 219 countries, between 1% and 2% in further 69 countries and greater than 25% in 76 countries.^108^ These figures are in stark contrast to the 2002–2003 severe acute respiratory syndrome coronavirus, SARS‐CoV‐1, which infected approximately 8000 people with a case fatality rate of approximately 9.5%.^76^ These differences in case numbers illustrate the higher efficient transmission of SARS‐CoV‐2 than SARS‐CoV1 by way of nasopharyngeal viral shedding at a time when symptoms are mild.^34^ That efficiency is a direct consequence of a nasopharyngeal tropism for SARS‐CoV‐2 that collectively involves the interplay between entropy and enthalpy, which results in the following:

‐ a higher affinity for the ACE2 target,
‐ the effective passage across the mucosal bilayer gradient,
‐ the enhanced efficiency of target cell entry mediated by furin at the viral polybasic cleavage at the junction of S1 and S2, and
‐ the inhibition of type I IFN inflammatory responses to allow the virus to actively infect and replicate in bystander cells.

The population‐based figures also indicate that although the case fatality rates for SARS‐CoV‐2 are lower than those for SARS‐CoV1, the total global mortality is very much higher because of the higher efficiency of transmission of SARS‐CoV‐2. The population‐based data are informative in the context of the thermodynamic underpinnings of the disease. It has been estimated that at the peak of infection, each infected person carries 10^9^ to 10^11^ virions with a total mass of between 1µg and 100 µg^109^ and based on the 226.8 million confirmed cases of COVID‐19 disease in September 2021, would reflect a circulating human host SARS‐CoV‐2 mass in the range of 0.2 to 20 kg. This entire viral mass is a consequence of millions of binding events involving SARS‐CoV‐2 with its ACE2 target and the appropriation of the host constituent molecules to assemble virions. Each of these binding events, as discussed earlier, is described by the GFE considerations that determine the stability, binding affinity of the SARS‐CoV‐2 and binding energy that is reflected in the thermodynamic spontaneity.

Accordingly, it is not unreasonable to assume that the epidemiological characteristics of the COVID‐19 pandemic must, in part or fully reflect the fundamental characteristics of SARS‐CoV‐2 target binding, assembly, and organization that occurs in a single cell, tissues or collectively in the human. Support for this view comes from several sources. Firstly, Ghanbari et al. assumed that the spread of COVID‐19 is a thermodynamic system focused on entropy (a measure of the disorder of a system) and used this to predict behavior and model COVID‐19 propagation.^110^ Secondly, Lucia et al., building on the thermodynamics of complex systems, also focused on entropy as the function to determine the evolution of the infectious disease and the time of spread.^111^ They reasoned that the spread of infection can be examined as an open thermodynamic system and in doing so, they focused only on the Gibbs entropy shape. Moreover, they were able to demonstrate that the model has been confirmed for the COVID‐19 pandemic.

What is apparent from these considerations is that the measure of entropy predicts outcomes at a population level – it is the thermodynamic interplay between entropy and enthalpy that governs the spontaneity of infection of a single cell with SARS‐CoV‐2.

## 9 HOW DO THERMODYNAMIC SPONTANEITY, MASS ACTION, AND TROPISM PROVIDE THE FUNDAMENTAL PLATFORM FOR TREATING THE HOST IN THE COVID‐19 DISEASE?

There has been a significant focus on identifying therapeutic agents that would be of benefit in treating patients with COVID‐19 disease. Generally, this has often involved identifying on or off‐target (repurposed) existing and approved drugs that may be useful in COVID‐19. This has been well described by Sultana et al., where they classified the approaches into those that inhibit key steps in the SARS‐CoV‐2 life cycle (viral replication, virion assembly/release) or those that counteract the effects of infection and the attending inflammation (anti‐inflammatory and immunomodulating drugs).^112^ This is an important area that is to be encouraged and one on which we have commented upon previously.^113^, ^114^ The one cautionary area that we suggest needs further discussion relates to the initial dampened IFN response with the suppressed immune response in the nasal passage at the very commencement of infection. It could be argued that this initial dampened inflammatory response facilitates viral replication, bystander cell spread and viral shedding. It follows that further suppression of inflammation through therapeutics may not achieve the desired outcome at this initial stage of infection but may do so immediately after symptoms become apparent.

An advantage in exploring the thermodynamic spontaneity, mass action, and tropism of SARS‐CoV‐2 in COVID‐19 disease is that it has the potential to highlight the areas of vulnerability of this virus and to identify areas of intervention. From the considerations discussed in this review, the passage of SARS‐CoV‐2, from its shedding from the epithelium in the nasopharyngeal tissues of an infected individual to its attachment to ACE2 on the nasal epithelial cells in a new host, represents an area of viral vulnerability with respect to infectivity. It is self‐evident that social isolation and the wearing of masks interferes with the aerosol mediated passage of the virus from infected individual to new host. An additional important approach has been suggested by Hou et al. and involves therapeutic strategies that decrease viral titers in the nasal tissue early in the progression of the disease.^82^ The key focus of this type of intervention is to prevent viral seeding of the lower respiratory tract with its attending serious disease. In a similar fashion, Bridges et al. have suggested that because the ciliated cells in the sino‐nasal airway are an initial infection site, this is where treatments should be designed to block infection and limit viral propagation.^96^ Additionally, Hou et al. have suggested strategies that involve nasal lavages, topical antivirals, or immune modulation.^82^

Although it is beyond the scope of this review to detail the potential therapeutic approaches, the role of mucosal IgA deserves comment. IgA protects the epithelial cell barriers from pathogens and is active against rotavirus, poliovirus, influenza virus and SARS‐CoV‐2.^115^ As pointed out by Russell et al., the first interactions that occur between SARS‐CoV‐2 and the immune system must occur at the respiratory mucosal surface, and they propose there is a significant role for mucosal immunity and for secretory as well as circulating IgA antibodies.^79^

We hypothesize that the upper respiratory tract nasopharyngeal mucosal interface may represent a potential novel therapeutic and immunological target for preventing progression to serious disease in COVID‐19.

## 10 SUMMARY AND CONCLUSIONS

**FIGURE 7:**
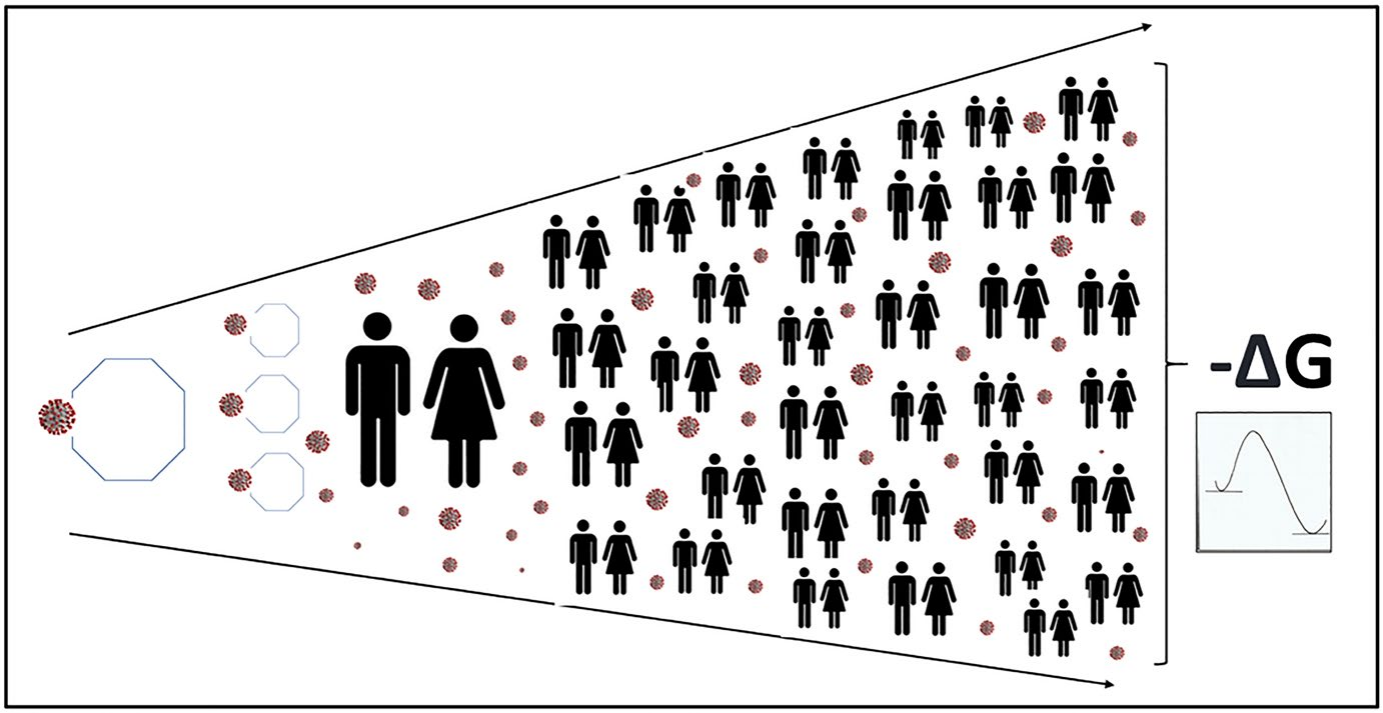
Epidemiological characteristics of the COVID‐19 pandemic reflect repeated SARS‐CoV‐2 target binding at enormous scale. Illustrates how entropy considerations predict outcomes not only at a cellular level but across infected populations reflecting the enormity of the repetition scale of thermodynamic spontaneity associated with the high affinity of SARS‐CoV‐2 for the ACE2 target in single cells, in multiple cells, in organs and tissues and across multiple cells in populations

The passage of SARS‐CoV‐2 within humans and across human populations displays the key characteristics of a complex system that underpins COVID‐19 disease. This complex system is powerfully described by the thermodynamic considerations underpinning the physiological and pharmacological properties involved in SARS‐CoV‐2, from infection of a single cell to its global spread (Figure 7). It is the synchrony of the simplicity of the viral surface change together with the liberation of GFE causing thermodynamic spontaneity that fundamentally drives COVID‐19 disease.

This understanding of thermodynamic spontaneity, mass action, and tropism provides the key platform to observe and understand the severity of disease that SARS‐CoV‐2 has on a human. This platform enables an understanding of the core reasons for enhanced infectivity with emerging variants and observes the parallel evolutionary convergence seen with SARS‐CoV‐2 and other infective viruses such as influenza. It provides the rational explanation for the time course of the pathophysiology in COVID‐19. Finally, this platform seeks out the areas of vulnerability of the virus for therapeutic intervention as it proceeds on a path of infectivity. Above all else, this analysis has highlighted the clash of two distinct evolutionary paths that of the vulnerable complexity of the human with that of lethal simplicity of SARS‐CoV‐2. It is clear this pandemic is all about a lethality driven by the fundamental laws of thermodynamics.

## DISCLOSURE

The authors have no conflicts of interest to declare.

## FUNDING INFORMATION

No funding was received for this article

## AUTHOR CONTRIBUTIONS

Richard J. Head conceived of the idea of investigating the role of thermodynamics and affinity measurements in the spread of SARS‐CoV‐2 from a single cell to a global population. Richard J. Head drafted the manuscript with input from Eugenie R. Lumbers and Jennifer H. Martin. All authors made substantial contributions as part of a COVID‐19 systems analysis collective.

## Data Availability

Not applicable.
